# Convergent Synthesis of Polysubstituted Furans via Catalytic Phosphine Mediated Multicomponent Reactions

**DOI:** 10.3390/molecules24244595

**Published:** 2019-12-16

**Authors:** Xia Fan, Rongshun Chen, Jie Han, Zhengjie He

**Affiliations:** 1 The State Key Laboratory of Elemento-Organic Chemistry, College of Chemistry, Nankai University, Tianjin 300071, China; fanxia0124@163.com (X.F.); hanjie@nankai.edu.cn (J.H.); 2 Jiangsu Key Laboratory of Pesticide Science, College of Sciences, Nanjing Agricultural University, Nanjing 210095, China

**Keywords:** polysubstituted furans, multicomponent reaction, silanes, phosphine oxides, catalytic Wittig reaction

## Abstract

Tri- or tetrasubstituted furans have been prepared from terminal activated olefins and acyl chlorides or anhydrides by a multicomponental convergent synthesis mode. Instead of stoichiometric *n*Bu_3_P, only catalytic *n*Bu_3_P or *n*Bu_3_P=O is needed to furnish the furans in modest to excellent yields with a good functional group tolerance under the aid of reducing agent silane. This synthetic method features a silane-driven catalytic intramolecular Wittig reaction as a key annulation step and represents the first successful application of catalytic Wittig reaction in multicomponent cascade reaction.

## 1. Introduction

The Wittig reaction [1,2,3,4] provides a powerful tool for convenient construction of carbon-carbon double bonds in synthetic chemistry. Despite the popularity of this reaction, some major drawbacks are associated with the formation of stoichiometric phosphine oxides as by-products. The troublesome purification of the by-products [5] makes them valueless on an industrial scale [6,7,8]. Also, most well-designed phosphines, especially chiral phosphine reagents [9,10,11,12,13,14,15], are very expensive and high cost hampers their applications in asymmetric Wittig reaction [16,17,18,19,20,21,22,23,24,25]. In this context, it is highly desired to develop a catalytic version of phosphine-mediated Wittig reaction by in-situ recycling by-product phosphine oxide under the aid of a reducing agent. However, it is a challenging task to reuse phosphine oxides to effect a catalytic Wittig reaction [26,27,28]. First, phosphine oxides are not generally easy to be reduced into the corresponding phosphines at a substantially fast rate under mild conditions; second, the employed reducing agent should be safe for other functional groups of the substrates [29,30]. Consequently, although many new methods for reduction of phosphine oxides have emerged by using reducing agents such as DIBAL-H [31,32], LiAlH_4_ [33,34], boron compounds [35,36,37,38], and others [39,40,41,42,43,44,45], applicable ones for catalytic Wittig reactions remain rare. Recently, silanes have been recognized as powerful reducing agents for in-situ deoxygenation of phosphine oxides with good functional tolerance after a series of successful applications of silanes in the transformation of a stoichiometric phosphine-mediated reaction into a catalytic one [46,47,48,49,50,51,52]. In 2009, O’Brien et al. [53] developed the first catalytic Wittig reaction for efficient synthesis of alkenes in moderate to high yields by using silanes such as diphenyl silane or trimethoxysilane to reuse in-situ by-product phosphine oxide [54,55,56]. In 2012, Beller and coworkers realized efficient and chemoselective reduction of tertiary and secondary phosphine oxides to their corresponding phosphines by using silanes under the aid of catalytic copper complexes [57] or specific Brønsted acids [58]. Under the reported conditions, various reducible functional groups such as ketones, aldehydes, olefins, nitriles, and esters were well tolerated. Based on these encouraging findings, a number of important stoichiometric phosphine-involved reactions, including (aza-)Wittig [59,60,61,62,63,64,65,66,67,68,69,70,71,72,73,74,75], Mitsunobu [76,77,78], Staudinger [79,80,81,82,83,84], Appel [85,86], Cadogan [87], and others [88,89,90,91,92,93] have been successfully developed into the catalytic phosphine mediated ones.

**Scheme 1:**
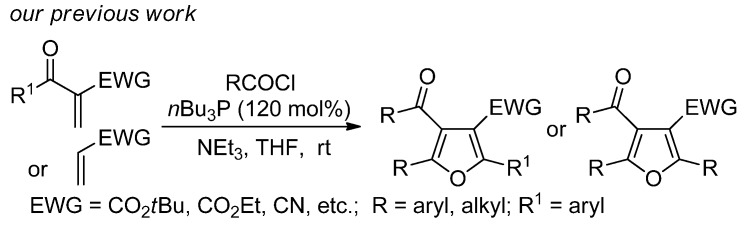
Phosphine-mediated synthesis of tetra-substituted furans.

Multicomponent reactions (MCRs) [94,95,96] have attracted much interest from organic chemists in recent years as a highly efficient synthetic strategy. In 2012, our group [97] realized a stoichiometric phosphine-mediated pseudo-three-component reaction between terminal activated olefins and acyl chlorides or anhydrides, leading to a convenient and convergent synthesis of tetra-substituted furans in moderate to excellent yields. The highly functionalized furan product was formed from one molecule of activated olefin and two or three molecules of acyl chloride or anhydride under the mediation of *n*Bu_3_P through a multiple domino sequence consisting of *C*-acylation, *O*-acylation, and intramolecular Wittig reaction (Scheme 1). A concomitant by-product *n*Bu_3_P=O was also formed [97]. Considering its high efficiency in synthesis of tetra-substituted furans and its characteristic of the multicomponent cascade reaction, we were interested in exploring a catalytic version of this reaction by using silanes as reducing agents. As mentioned above, although silanes have been successfully used as reducing agents in a series of catalytic versions of important phosphine-mediated reactions like the Wittig reaction [53,54,55,56,57,58,59,60,61,62,63,64,65,66,67,68,69,70,71,72,73,74,75,76,77,78,79,80,81,82,83,84,85,86,87,88,89,90,91,92,93], this silane-reduction strategy has never been applied to a stoichiometric phosphine mediated multicomponent cascade reaction before. Furthermore, the addition of reducing agent silane will certainly result in an increased complexity of a multiple domino reaction sequence. Herein we report the relevant results from such challenging studies.

## 2. Results and Discussion

Our investigation started with a model reaction between *tert*-butyl acrylate **1a** and 3-chlorobenzoyl chloride **2a** (Table 1). Under the similar conditions of our previous work [97], *tert*-butyl acrylate (0.5 mmol) and 3-chlorobenzoyl chloride (1.8 mmol) were added to THF (2.0 mL) under N_2_, followed by addition of catalytic *n*Bu_3_P (0.1 mmol), NEt_3_ (2.7 mmol) and Ph_2_SiH_2_ (0.6 mmol), the resulting mixture was stirred at room temperature for 24 h. The desired product **3aa** was obtained, but only in 11% yield (entry 1). The reaction was then conducted at 60 °C and the yield of **3aa** was not substantially improved (entry 2). Although only a trace amount of the product was observed in a PhSiH_3_ mediated reaction run at room temperature in toluene (entry 3), to our delight, tetra-substituted furan **3aa** was obtained in 74% yield when the temperature was raised to 110 °C in refluxing toluene (entry 4). Other silanes such as Ph_2_SiH_2_, (MeO)_3_SiH, polymethylhydrosiloxane (PMHS) and Ph_3_SiH were effective but offered inferior results (entries 5‒9); SiHCl_3_ were ineffective at all (entry 10). The loading amount of PhSiH_3_ was studied and a decreased loading (0.4 mmol) could also smoothly offer a yield of 84% (entries 11‒13). A lower reaction temperature (80 °C) resulted in a decreased yield (entry 14). Solvents like dioxane, xylene, and DMF only gave inferior results and acetonitrile was detrimental to the reaction (entries 15‒18). A lowered loading amount of *n*Bu_3_P (0.05 mmol, 10 mol%) was investigated, only giving the product in a moderate yield (entry 19). Other phosphines such as PPh_3_ and Ph_2_PMe were totally ineffective as reported before [97]. Thus, the optimized conditions were established as those shown in Table 1, entry 12.

Under the optimized conditions, the substrate scope of alkenes **1** and acyl chlorides **2** were further examined (Table 2). Gratifyingly, reducible functional groups such as ketone, acyl chloride, olefin, nitro, cyano, and ester were all well tolerated in this multicomponent reaction, and moderate to excellent yields were generally obtained when different substrates were employed (Table 2). When *tert*-butyl acrylate **1a** was employed, a series of substituted benzoyl chlorides **2**, except *o*-chlorobenzoyl chloride **2c** and 4-nitrobenzoyl chloride **2f**, readily afforded the corresponding tetra-substituted furans **3** in good yields (entries 1‒6). Hetero-aryl acyl chloride like 2-thiofuroyl chloride (**2g**) was also effective in this transformation, furnishing the corresponding tetrasubstituted furan **3ag** in moderate yield (entry 7). Methyl, ethyl, *n*-butyl and benzyl acrylates (**1b**–**e**), acrylonitrile **1f** were all good candidates for this reaction and the expected products were readily delivered in moderate to good yields (entries 8‒17). Aliphatic anhydride **2a’** was also found to be a suitable substrate, albeit giving a lower yield (entry 18).

**Scheme 2:**
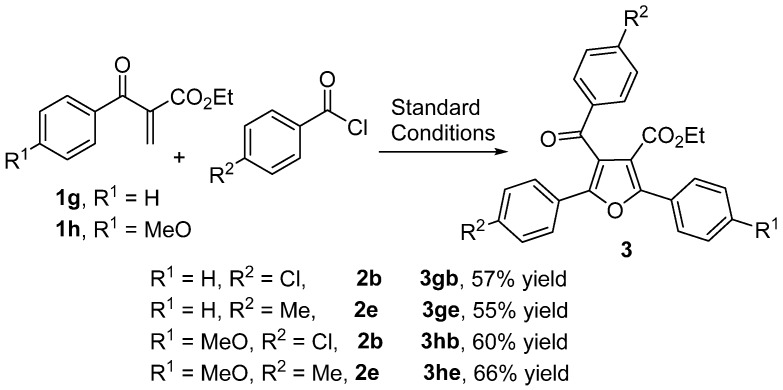
Synthesis of tetra-substituted furans **3** from di-substituted alkene **1g** and **1h**.

To further test the generality of the reaction, di-substituted alkene **1g** and **1h** were also studied (Scheme 2). We found that α,β-unsaturated ketones were well compatible under standard conditions, affording the corresponding tetra-substituted furans in satisfactory yields with different benzoyl chlorides (Scheme 2).

**Scheme 3:**
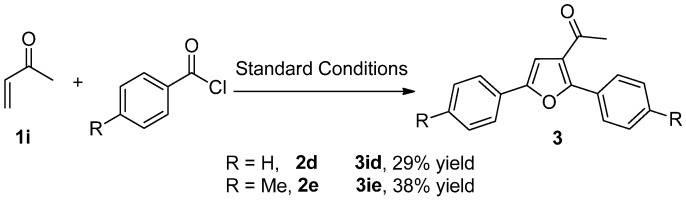
Synthesis of tri-substituted furans **3** from methyl vinyl ketone **1i**.

To our surprise, methyl vinyl ketone (MVK) **1i** was also effective under the catalytic conditions, furnishing the corresponding tri-substituted furans **3id** and **3ie** in fair yields in the reactions with benzoyl chlorides (Scheme 3). It is worthy to note that, in our previous work [97], the more reactive alkene MVK **1i** failed to deliver the expected furans in appreciable yields under the mediation of stoichiometric *n*Bu_3_P. Presumably, under the silane-driven catalytic conditions, the instant concentration of *n*Bu_3_P would be kept at a relatively low level in the reaction mixture. The low concentration of *n*Bu_3_P may diminish the possible side reactions of the highly reactive olefin like **1i**.

**Scheme 4:**
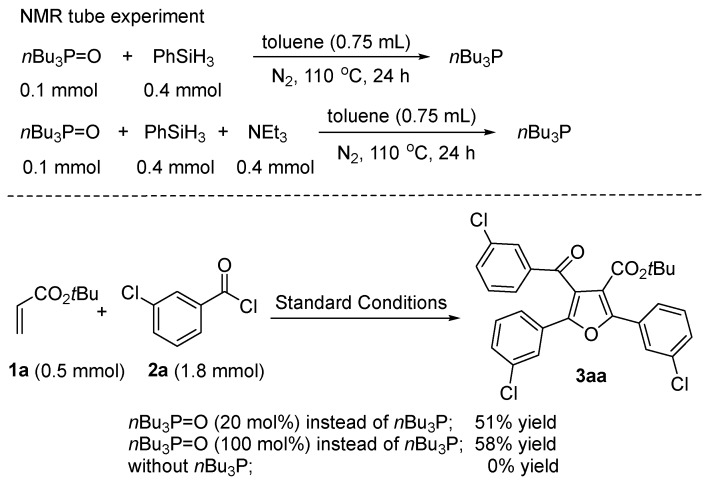
Control experiments.

In order to gain more insights about the reaction, some control experiments were conducted (Scheme 4). In a ^31^P NMR tracking experiment (a sealed capillary containing C_6_D_6_ was used for field locking and shimming), a signal at δ −32.4 ppm was observed which was identified as *n*Bu_3_P when *n*Bu_3_P=O (0.1 mmol) and silane PhSiH_3_ (0.4 mmol) were mixed with or without NEt_3_ (0.4 mmol) in toluene in an NMR tube under N_2_ for 24 h (for details, see Supporting Information). These results provided direct evidence for the regeneration of the *n*Bu_3_P catalyst. As expected, tetra-substituted furan **3aa** was readily obtained in 51% isolated yield when *n*Bu_3_P=O (20 mol%) was used instead of *n*Bu_3_P. In contrast, an increased loading of *n*Bu_3_P=O (100 mol%) only brought in a modestly improved yield (58%). The silane PhSiH_3_ alone could not effect the domino reaction. These results indicated that *n*Bu_3_P=O was the equivalent catalyst of *n*Bu_3_P in the presence of silane PhSiH_3_. It is noticeable that *n*Bu_3_P=O was inactive as a catalyst in Lin’s report [68].

**Scheme 5:**
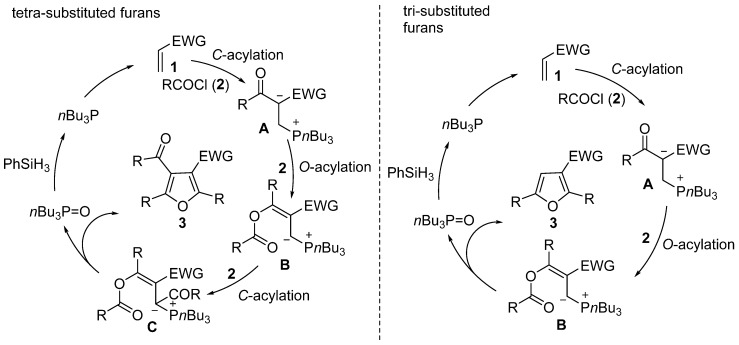
Proposed mechanism.

Based on the results of this work and previous reports [59,60,61,62,63,64,65,66,67,68,69,70,71,72,73,74,75,97], a multiple domino process is proposed to rationalize the formation of tetra-substituted furans **3** (Scheme 5, left). The catalytic cycle starts with the nucleophilic addition of *n*Bu_3_P to an activate alkene **1** like acrylates, the resulting intermediate undergoes a *C*-acylation reaction with acyl chloride **2** to give a phosphonium enolate A. The phosphorus ylide B, generated through the *O*-acylation reaction of intermediate A with acyl chloride 2 in the presence of NEt_3_, engages in another *C*-acylation reaction to deliver an ylide C under the aid of base NEt_3_. Finally, a polysubstituted furan is delivered through an intramolecular Wittig reaction of ylide C with the release of *n*Bu_3_P=O. The phosphine oxide *n*Bu_3_P=O is then in-situ reduced by silane PhSiH_3_ into *n*Bu_3_P, which enters the next catalytic cycle. Regarding the formation of the tri-substituted furans **3id** and **3ie** from MVK **1i**, a similar triple domino sequence of *C*-acylation/*O*-acylation/intramolecular Wittig reaction presumably occurs (Scheme 5, right).

## 3. Experimental Section

Unless otherwise noted, all reactions were carried out in a nitrogen atmosphere under anhydrous conditions. Solvents were purified prior to use according to standard procedures. ^1^H and ^13^C NMR spectra were recorded in CDCl_3_ with tetramethylsilane (TMS) as the internal standard. Column chromatography was performed on silica gel (200–300 mesh) using a mixture of petroleum ether (60–90 °C)/ethyl acetate as the eluant. 2-Acyl acrylates **1g**–**h** were prepared according to the reported procedure [98].

**Typical procedure for synthesis of highly functionalized furans 3:** Under a N_2_ atmosphere, to a solution of activated olefins **1a**–**1i** (0.5 mmol), acylation agent **2** (1.8 mmol) in toluene (2.0 mL) were sequentially added *n*Bu_3_P (25 μL, 0.1 mmol), Et_3_N (2.7 mmol) and silane (0.4 mmol) by means of microsyringe. The resulting reaction mixture was stirred at 110 °C for 24 h. After completion of the reaction as monitored by TLC, water (10 mL) was added. The mixture was extracted with CH_2_Cl_2_ (3 × 10 mL). The combined organic layer was washed with saturated brine (10 mL) and dried over anhydrous sodium sulfate. After filtration, the solvent was removed on a rotary evaporator under reduced pressure, and the residue was subjected to column chromatographic isolation on silica gel (eluted with petroleum ether/ ethyl acetate (40:1–20:1) to give furans **3**.

**3aa**–**3de**, **3fb**–**3he** are known compounds in our previous report [97]. NMR spectra of compounds **3** are available in Supplementary Materials.

**3aa**, 221 mg, 84% yield; as a yellow solid; m.p. 110–111 °C; ^1^H-NMR (400 MHz, CDCl_3_) δ 8.04 (d, *J* = 0.7 Hz, 1H), 7.98 (t, *J* = 1.7 Hz, 1H), 7.97–7.92 (m, 1H), 7.83 (d, *J* = 7.8 Hz, 1H), 7.67 (d, *J* = 1.7 Hz, 1H), 7.60–7.53 (m, 1H), 7.48–7.38 (m, 4H), 7.31–7.22 (m, 2H), 1.23 (s, 9H).

**3ab**, 199 mg, 75% yield; as a yellow solid; m.p. 198–199 °C; ^1^H-NMR (400 MHz, CDCl_3_) δ 8.00 (d, *J* = 8.7 Hz, 2H), 7.92 (d, *J* = 8.6 Hz, 2H), 7.53 (d, *J* = 8.7 Hz, 2H), 7.46 (dd, *J* = 8.7, 6.7 Hz, 4H), 7.30 (d, *J* = 8.7 Hz, 2H), 1.19 (s, 9H).

**3ac**, 87 mg, 33% yield; as a yellow solid; m.p. 118–120 °C; ^1^H-NMR (400 MHz, CDCl_3_) δ 7.66 (dd, *J* = 7.6, 1.5 Hz, 1H), 7.60 (dd, *J* = 7.4, 1.9 Hz, 1H), 7.53–7.46 (m, 2H), 7.42–7.34 (m, 2H), 7.34–7.27 (m, 3H), 7.27–7.24 (m, 1H), 7.23–7.16 (m, 2H), 1.22 (s, 9H).

**3ad**, 149 mg, 70% yield; as a white solid; m.p. 181–183 °C; ^1^H-NMR (400 MHz, CDCl_3_) δ 8.14–7.94 (m, 4H), 7.69–7.62 (m, 2H), 7.57 (t, *J* = 7.4 Hz, 1H), 7.53–7.41 (m, 5H), 7.37–7.26 (m, 3H), 1.16 (s, 9H).

**3ae**, 210 mg, 90% yield; as a white solid; m.p. 160–162 °C; ^1^H-NMR (400 MHz, CDCl_3_) δ 7.84 (d, *J* = 8.2 Hz, 2H), 7.79 (d, *J* = 8.1 Hz, 2H), 7.41 (d, *J* = 8.2 Hz, 2H), 7.15 (d, *J* = 8.1 Hz, 2H), 7.11 (d, *J* = 8.0 Hz, 2H), 6.97 (d, *J* = 8.1 Hz, 2H), 2.27 (s, 3H), 2.25 (s, 3H), 2.15 (s, 3H), 1.06 (s, 9H).

**3af**, 102 mg, 37% yield; as a yellow solid; m.p. 178–180 °C; ^1^H-NMR (400 MHz, CDCl_3_) δ 8.37 (t, *J* = 9.6 Hz, 4H), 8.30 (d, *J* = 8.7 Hz, 2H), 8.23 (d, *J* = 8.6 Hz, 2H), 8.18 (d, *J* = 8.5 Hz, 2H), 7.79 (d, *J* = 8.6 Hz, 2H), 1.22 (s, 9H).

**3ag**, 101 mg, 46% yield; as a oil; ^1^H-NMR (400 MHz, CDCl_3_) δ 8.10 (dd, *J* = 3.8, 1.0 Hz, 1H), 7.69 (dd, *J* = 4.9, 1.0 Hz, 1H), 7.61–7.59 (m, 1H), 7.48 (dd, *J* = 5.0, 1.0 Hz, 1H), 7.45 (dd, *J* = 3.7, 1.0 Hz, 1H), 7.32 (dd, *J* = 5.0, 1.0 Hz, 1H), 7.15 (dd, *J* = 5.0, 3.9 Hz, 1H), 7.09 (dd, *J* = 4.8, 3.9 Hz, 1H), 7.02 (dd, *J* = 5.0, 3.8 Hz, 1H), 1.25 (s, 9H).

**3bb**,196 mg, 81% yield; as a yellow solid; m.p. 185–186 °C; ^1^H-NMR (400 MHz, CDCl_3_) δ 7.99 (d, *J* = 8.4 Hz, 2H), 7.87 (d, *J* = 8.3 Hz, 2H), 7.55 (d, *J* = 8.4 Hz, 2H), 7.51–7.41 (m, 4H), 7.31 (d, *J* = 8.4 Hz, 2H), 3.50 (s, 3H).

**3be**, 190 mg, 84% yield; as a yellow solid; m.p. 185–186 °C; ^1^H-NMR (400 MHz, CDCl_3_) δ 7.93 (d, *J* = 8.0 Hz, 2H), 7.85 (d, *J* = 7.9 Hz, 2H), 7.53 (d, *J* = 8.0 Hz, 2H), 7.28 (d, *J* = 7.9 Hz, 2H), 7.22 (d, *J* = 7.8 Hz, 2H), 7.10 (d, *J* = 7.9 Hz, 2H), 3.46 (s, 3H), 2.41 (s, 3H), 2.37 (s, 3H), 2.29 (s, 3H).

**3cb**, 178 mg, 71% yield; as a white solid; m.p. 177–178 °C; ^1^H-NMR (400 MHz, CDCl_3_) δ 8.01 (d, *J* = 8.4 Hz, 2H), 7.90 (d, *J* = 8.3 Hz, 2H), 7.54 (d, *J* = 8.4 Hz, 2H), 7.45 (t, *J* = 9.2 Hz, 4H), 7.31 (d, *J* = 8.4 Hz, 2H), 4.00 (q, *J* = 7.1 Hz, 2H), 0.93 (t, *J* = 7.1 Hz, 3H).

**3ce**, 195 mg, 84% yield; as a white solid; m.p. 134–136 °C; ^1^H-NMR (400 MHz, CDCl_3_) δ 7.95 (d, *J* = 7.9 Hz, 2H), 7.88 (d, *J* = 7.8 Hz, 2H), 7.53 (d, *J* = 7.9 Hz, 2H), 7.28 (d, *J* = 7.9 Hz, 2H), 7.23 (d, *J* = 7.9 Hz, 2H), 7.10 (d, *J* = 7.9 Hz, 2H), 3.97 (q, *J* = 7.0 Hz, 2H), 2.41 (s, 3H), 2.38 (s, 3H), 2.29 (s, 3H), 0.90 (t, *J* = 7.1 Hz, 3H).

**3db**, 224 mg, 85% yield, as a yellow solid; m.p. 142–143 °C; ^1^H-NMR (400 MHz, CDCl_3_) δ 8.01 (d, *J* = 8.6 Hz, 2H), 7.90 (d, *J* = 8.5 Hz, 2H), 7.53 (d, *J* = 8.6 Hz, 2H), 7.44 (t, *J* = 8.6 Hz, 4H), 7.29 (d, *J* = 8.6 Hz, 2H), 3.96 (t, *J* = 6.6 Hz, 2H), 1.28–1.23 (m, 2H), 1.09–1.07 (m, 2H), 0.77 (t, *J* = 7.3 Hz, 3H).

**3de**, 193 mg, 83% yield, as a white solid; m.p. 120–121 °C; ^1^H-NMR (400 MHz, CDCl_3_) δ 7.95 (d, *J* = 8.1 Hz, 2H), 7.88 (d, *J* = 8.0 Hz, 2H), 7.51 (d, *J* = 8.1 Hz, 2H), 7.27 (d, *J* = 8.1 Hz, 2H), 7.22 (d, *J* = 8.0 Hz, 2H), 7.09 (d, *J* = 8.1 Hz, 2H), 3.93 (t, *J* = 6.5 Hz, 2H), 2.40 (s, 3H), 2.37 (s, 3H), 2.28 (s, 3H), 1.35–1.15 (m, 2H), 1.07–1.05 (m, 2H), 0.74 (t, *J* = 7.3 Hz, 3H).

**3ed**, 200 mg, 87% yield, as a white solid; m.p. 124–126 °C; ^1^H-NMR (400 MHz, CDCl_3_) δ 8.07 (dd, *J* = 6.5, 2.7 Hz, 2H), 7.92 (d, *J* = 7.6 Hz, 2H), 7.68 (d, *J* = 7.9 Hz, 2H), 7.57 (t, *J* = 7.4 Hz, 1H), 7.53–7.47 (m, 2H), 7.41 (t, *J* = 7.7 Hz, 2H), 7.38–7.31 (m, 3H), 7.30–7.22 (m, 2H), 7.05 (d, *J* = 7.1 Hz, 2H), 5.00 (s, 2H); ^13^C NMR (101 MHz, CDCl_3_) δ 192.2, 162.2, 156.3, 150.1, 137.3, 134.6, 133.4, 129.9, 129.3, 128.9, 128.8, 128.7, 128.6, 128.5, 128.3, 128.2, 128.2, 128.0, 125.9, 121.8, 115.1, 66.7. HRMS-ESI calcd. for C_31_H_23_O_4_ [M + H]^+^ 459.1591; found 459.1593.

**3fb**, 85 mg, 38% yield, as a yellow solid; m.p. 181–182 °C; ^1^H-NMR (400 MHz, CDCl_3_) 8.03 (d, *J* = 8.6 Hz, 2H), 7.81 (d, *J* = 8.5 Hz, 2H), 7.52 (dd, *J* = 8.3, 6.2 Hz, 4H), 7.41 (d, *J* = 8.5 Hz, 2H), 7.33 (d, *J* = 8.6 Hz, 2H).

**3fd**, 100 mg, 57% yield, as a white solid; m.p. 152–154 °C; ^1^H-NMR (400 MHz, CDCl_3_) δ 8.18–8.07 (m, 2H), 7.88 (d, *J* = 7.3 Hz, 2H), 7.62–7.48 (m, 6H), 7.39 (t, *J* = 7.7 Hz, 2H), 7.32 (t, *J* = 6.4 Hz, 3H).

**3fe**, 85 mg, 44% yield, as a yellow solid; m.p. 165–167 °C; ^1^H-NMR (400 MHz, CDCl_3_) δ 7.99 (d, *J* = 8.1 Hz, 2H), 7.80 (d, *J* = 8.0 Hz, 2H), 7.48 (d, *J* = 8.1 Hz, 2H), 7.31 (d, *J* = 8.0 Hz, 2H), 7.20 (d, *J* = 7.9 Hz, 2H), 7.10 (d, *J* = 8.0 Hz, 2H), 2.42 (s, 3H), 2.37 (s, 3H), 2.31 (s, 3H).

**3aa’**, 32 mg, 27% yield; as a oil; ^1^H-NMR (400 MHz, CDCl_3_) δ 2.46 (s, 3H), 2.43 (s, 3H), 2.34 (s, 3H), 1.55 (s, 9H).

**3gb**, 133 mg, 57% yield; as a white solid; m.p. 135–137 °C; ^1^H-NMR (400 MHz, CDCl_3_) δ 8.06–8.00 (m, 2H), 7.94–7.89 (m, 2H), 7.59–7.53 (m, 2H), 7.52–7.46 (m, 3H), 7.46–7.42 (m, 2H), 7.33–7.28 (m, 2H), 4.00 (q, *J* = 7.1 Hz, 2H), 0.94 (t, *J* = 7.1 Hz, 3H).

**3ge**, 117 mg, 55% yield; as a white solid; m.p. 128–130 °C; ^1^H-NMR (400 MHz, CDCl_3_) δ 8.09–8.02 (m, 2H), 7.88 (d, *J* = 8.2 Hz, 2H), 7.53 (d, *J* = 8.2 Hz, 2H), 7.51–7.41 (m, 3H), 7.24 (d, *J* = 8.1 Hz, 2H), 7.12 (d, *J* = 8.1 Hz, 2H), 3.97 (q, *J* = 7.1 Hz, 2H), 2.40 (s, 3H), 2.31 (s, 3H), 0.90 (t, *J* = 7.1 Hz, 3H).

**3hb**, 149 mg, 60% yield; as a yellow solid; m.p. 150–151 °C; ^1^H-NMR (400 MHz, CDCl_3_) δ 8.03 (d, *J* = 8.9 Hz, 2H), 7.91 (d, *J* = 8.5 Hz, 2H), 7.55 (d, *J* = 8.7 Hz, 2H), 7.43 (d, *J* = 8.6 Hz, 2H), 7.28 (d, *J* = 8.7 Hz, 2H), 7.00 (d, *J* = 8.9 Hz, 2H), 3.99 (q, *J* = 7.1 Hz, 2H), 3.87 (s, 3H), 0.92 (t, *J* = 7.1 Hz, 3H).

**3he**, 150 mg, 66% yield; as a white solid; m.p. 132–133 °C; ^1^H-NMR (400 MHz, CDCl_3_) δ 8.04 (d, *J* = 8.6 Hz, 2H), 7.87 (d, *J* = 7.8 Hz, 2H), 7.52 (d, *J* = 7.9 Hz, 2H), 7.23 (d, *J* = 8.0 Hz, 2H), 7.11 (d, *J* = 8.1 Hz, 2H), 7.00 (d, *J* = 8.8 Hz, 2H), 3.96 (q, *J* = 7.1 Hz, 2H), 3.87 (s, 3H), 2.40 (s, 3H), 2.30 (s, 3H), 0.89 (t, *J* = 7.1 Hz, 3H).

**3id**, 38 mg, 29% yield; as a yellow oil; ^1^H-NMR (400 MHz, CDCl_3_) δ 7.77–7.73 (m, 2H), 7.58 (dd, *J* = 7.8, 1.7 Hz, 2H), 7.44–7.35 (m, 2H), 7.28 (dd, *J* = 13.2, 5.4 Hz, 2H), 7.21–7.15 (m, 2H), 6.21 (d, *J* = 0.8 Hz, 1H), 2.31 (s, 3H); ^13^C NMR (101 MHz, CDCl_3_) δ 191.9, 154.5, 151.1, 138.2, 132.6, 130.0, 129.7, 128.6, 128.2, 128.1(overlap), 127.2, 121.7, 109.7, 13.4; HRMS-ESI calcd. for C_18_H_15_O_2_ [M + H]^+^ 263.1067; found 263.1071.

**3ie**, 55 mg, 38% yield; as a yellow oil; ^1^H-NMR (400 MHz, CDCl_3_) δ 7.75 (d, *J* = 8.1 Hz, 2H), 7.58 (d, *J* = 8.2 Hz, 2H), 7.17 (d, *J* = 8.0 Hz, 2H), 7.09 (d, *J* = 8.1 Hz, 2H), 6.24 (d, *J* = 0.8 Hz, 1H), 2.38 (s, 6H), 2.31 (s, 3H); ^13^C NMR (101 MHz, CDCl_3_) δ 191.7, 154.3, 150.6, 143.4, 138.5, 135.7, 129.8, 128.9 (overlap), 127.3, 127.0, 121.3, 109.7, 21.6, 21.3, 13.4; HRMS-ESI calcd. for C_20_H_19_O_2_ [M + H]^+^ 291.1380; found 291.1383.

## 4. Conclusions

In conclusion, a convergent synthetic method for tri- or tetra-substituted furans has been developed by catalytic phosphine mediated multicomponental cascade reactions. Instead of stoichiometric *n*Bu_3_P, only catalytic *n*Bu_3_P or *n*Bu_3_P=O is needed to deliver the furans in modest to excellent yields. A broad scope of substrates, bearing various reducible functional groups including ketone, acyl chloride, olefin, nitro, cyano, and ester, are all well tolerated in the presence of reducing agent silane. This synthetic method features a silane-driven catalytic intramolecular Wittig reaction as a key step and represents the first successful application of catalytic Wittig reaction in a multicomponent cascade reaction. Future efforts in our laboratory will be directed toward exploring the asymmetric reactions involving catalytic chiral phosphine mediated Wittig reaction, the results of which will be reported in due course.

## Figures and Tables

**Table 1:**
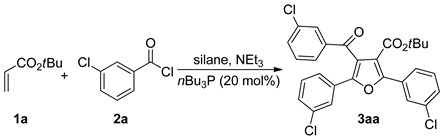
Survey of the model reaction conditions.^a.^

Entry	Silane (mmol)	Solvent	Temp (°C)	3 (%) ^b^
1	Ph_2_SiH_2_ (0.6)	THF	rt	11
2	Ph_2_SiH_2_ (0.6)	THF	60	12
3	PhSiH_3_ (0.6)	Toluene	rt	trace
4	PhSiH_3_ (0.6)	Toluene	110	74
5	Ph_2_SiH_2_ (0.6)	Toluene	110	42
6	(MeO)_3_SiH (0.6)	Toluene	110	33
7	PMHS (0.034)	Toluene	110	26
8	PMHS (0.106)	Toluene	110	30
9	Ph_3_SiH (1.2)	Toluene	110	18
10	SiHCl_3_ (0.6)	Toluene	rt	trace
11	PhSiH_3_ (0.25)	Toluene	110	65
12	PhSiH_3_ (0.4)	Toluene	110	84
13	PhSiH_3_ (0.75)	Toluene	110	49
14	PhSiH_3_ (0.4)	Toluene	80	56
15	PhSiH_3_ (0.4)	Dioxane	110	54
16	PhSiH_3_ (0.4)	Xylene	110	60
17	PhSiH_3_ (0.4)	DMF	110	11
18	PhSiH_3_ (0.4)	CH_3_CN	80	trace
19 ^c^	PhSiH_3_ (0.4)	Toluene	110	48

^a^ Typical conditions: a mixture of **1a** (0.5 mmol), **2a** (1.8 mmol), *n*Bu_3_P (0.1 mmol), NEt_3_ (2.7 mmol) and silane in the specified solvent (2.0 mL) under N_2_ at indicated temperature for 24 h. ^b^ Isolated yield based on **1a**. ^c^ The loading amount of *n*Bu_3_P was 0.05 mmol.

**Table 2:**
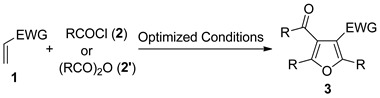
Synthesis of tetra-substituted furans **3** from olefins **1** and acyl chlorides **2** or anhydride **2′**.^a^

Entry	EWG in 1	R in 2 or 2′	3 (%) ^b^
1	CO_2_*t*Bu (**1a**)	3-ClC_6_H_4_ (**2a**)	**3aa**, 84
2	CO_2_*t*Bu (**1a**)	4-ClC_6_H_4_ (**2b**)	**3ab**, 75
3	CO_2_*t*Bu (**1a**)	2-ClC_6_H_4_ (**2c**)	**3ac**, 33
4	CO_2_*t*Bu (**1a**)	Ph(**2d**)	**3ad**, 70
5	CO_2_*t*Bu (**1a**)	4-MeC_6_H_4_ (**2e**)	**3ae**, 90
6	CO_2_*t*Bu (**1a**)	4-NO_2_C_6_H_4_ (**2f**)	**3af**, 37
7	CO_2_*t*Bu (**1a**)	2-thienyl (**2g**)	**3ag**, 46
8	CO_2_Me (**1b**)	4-ClC_6_H_4_ (**2b**)	**3bb**, 81
9	CO_2_Me (**1b**)	4-MeC_6_H_4_ (**2e**)	**3be**, 84
10	CO_2_Et (**1c**)	4-ClC_6_H_4_ (**2b**)	**3cb**, 71
11	CO_2_Et (**1c**)	4-MeC_6_H_4_ (**2e**)	**3ce**, 84
12	CO_2_*n*Bu (**1d**)	4-ClC_6_H_4_ (**2b**)	**3db**, 85
13	CO_2_*n*Bu (**1d**)	4-MeC_6_H_4_ (**2e**)	**3de**, 83
14	CO_2_Bn (**1e**)	Ph (**2d**)	**3ed**, 87
15	CN (**1f**)	4-ClC_6_H_4_ (**2b**)	**3fb**, 38
16	CN (**1f**)	Ph (**2d**)	**3fd**, 57
17	CN (**1f**)	4-MeC_6_H_4_ (**2e**)	**3fe**, 44
18	CO_2_*t*Bu (**1a**)	Me (**2′a**)	**3aa’**, 27

^a^ Optimized conditions: under a N_2_ atmosphere and at 110 °C, alkenes **1** (0.5 mmol) and acyl chlorides **2** or anhydride **2′** (1.8 mmol) were added into toluene (2.0 mL), followed by additions of *n*Bu_3_P (0.1 mmol), NEt_3_ (2.7 mmol) and PhSiH_3_ (0.4 mmol), the resulting mixture was stirred for 24 h. ^b^ Isolated yield based on **1**.

## Supplementary Materials

The supplementary materials are available online.
