# Supplementary material for: Convergent Synthesis of Polysubstituted Furans via Catalytic Phosphine Mediated Multicomponent Reactions
Source: Molecules. 2019 Dec 16;24(24):4595. doi: 10.3390/molecules24244595 (PMC6943692; doi:10.3390/molecules24244595)
Supplement: Supplementary file 1 [file molecules-24-04595-s001.pdf]

Supporting Information

Convergent Synthesis of Polysubstituted Furans via Catalytic Phosphine-Mediated Multicomponent Reactions

Xia Fan,[1] Rongshun Chen,[2,]* Jie Han,[1] Zhengjie He[1,]*

[1]*The State Key Laboratory of Elemento-Organic Chemistry, College of Chemistry, Nankai University, Tianjin 300071, China*

[2]*Jiangsu Key Laboratory of Pesticide Science, College of Sciences, Nanjing Agricultural University, Nanjing 210095, China*

e-mail: rongshunchen@njau.edu.cn; zhengjiehe@nankai.edu.cn

Table of Contents

I. ^{31}P NMR Tracking Experiments S2

II. NMR spectra for **3** S3

I. ^{31}P NMR Tracking Experiments

Experiment 1:

Under a N_2 atmosphere, *n*Bu_3P=O (0.1 mmol), $PhSiH_3$ (0.4 mmol) were added into toluene (0.75 mL) in an NMR tube. The resulting reaction mixture was heated at 110 ℃ for 24 h and then monitored by ^{31}P NMR measurement.

Experiment 2:

Under a N_2 atmosphere, *n*Bu_3P=O (0.1 mmol), $PhSiH_3$ (0.4 mmol) and NEt_3 (0.4 mmol) were added into toluene (0.75 mL) in an NMR tube. The resulting reaction mixture was heated at 110 ℃ for 24 h and then monitored by ^{31}P NMR measurement.

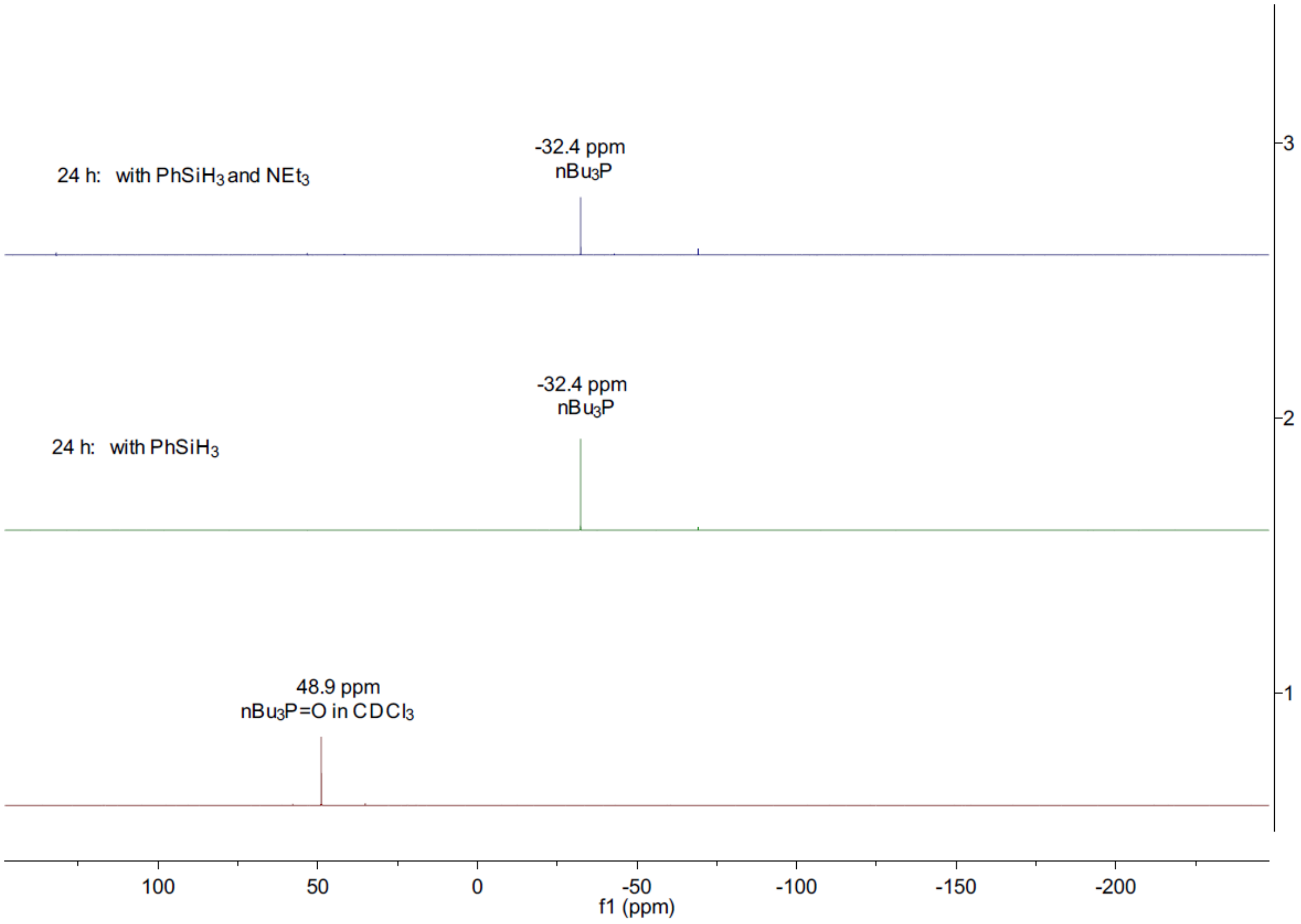

II. NMR Spectra for 3

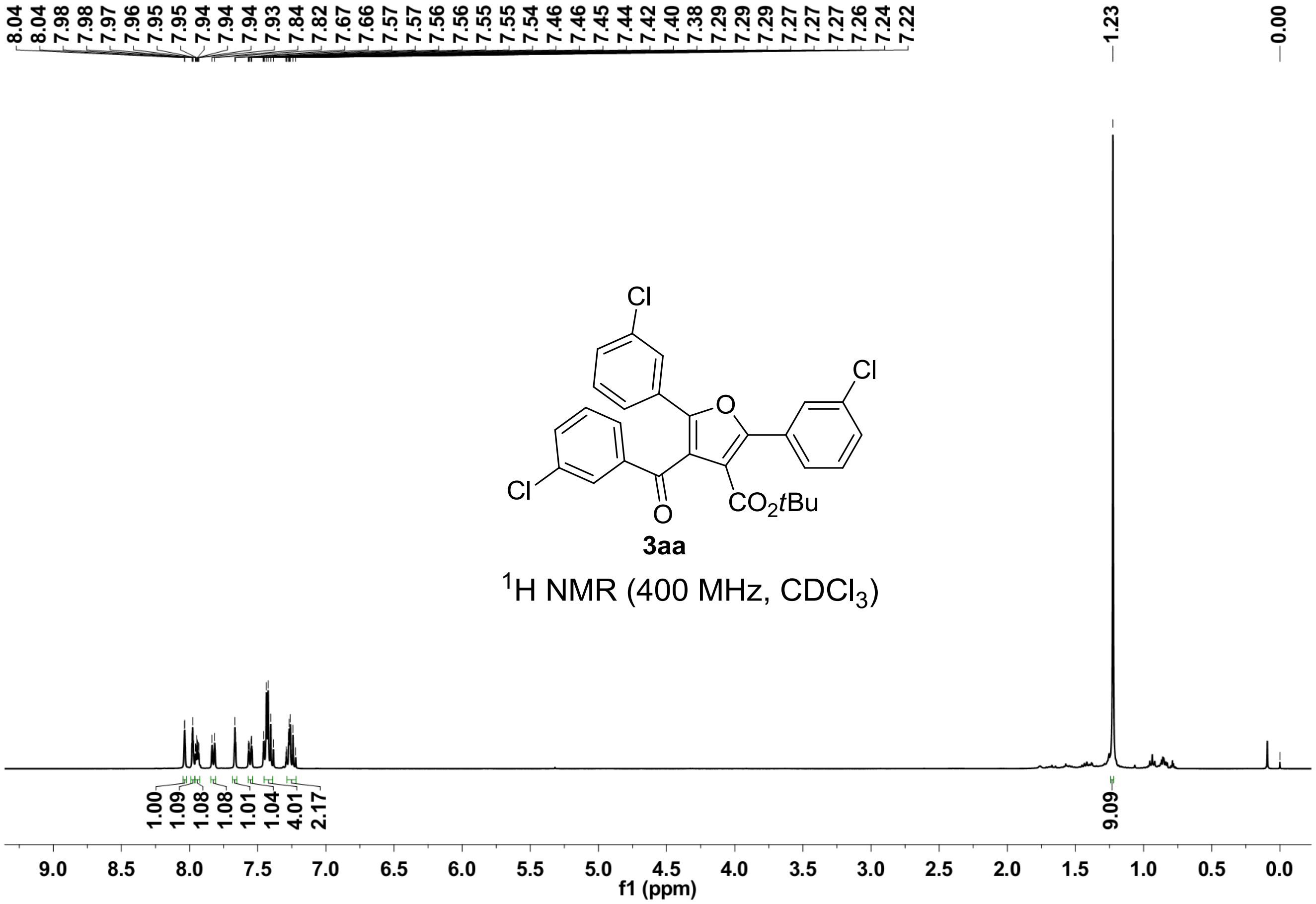

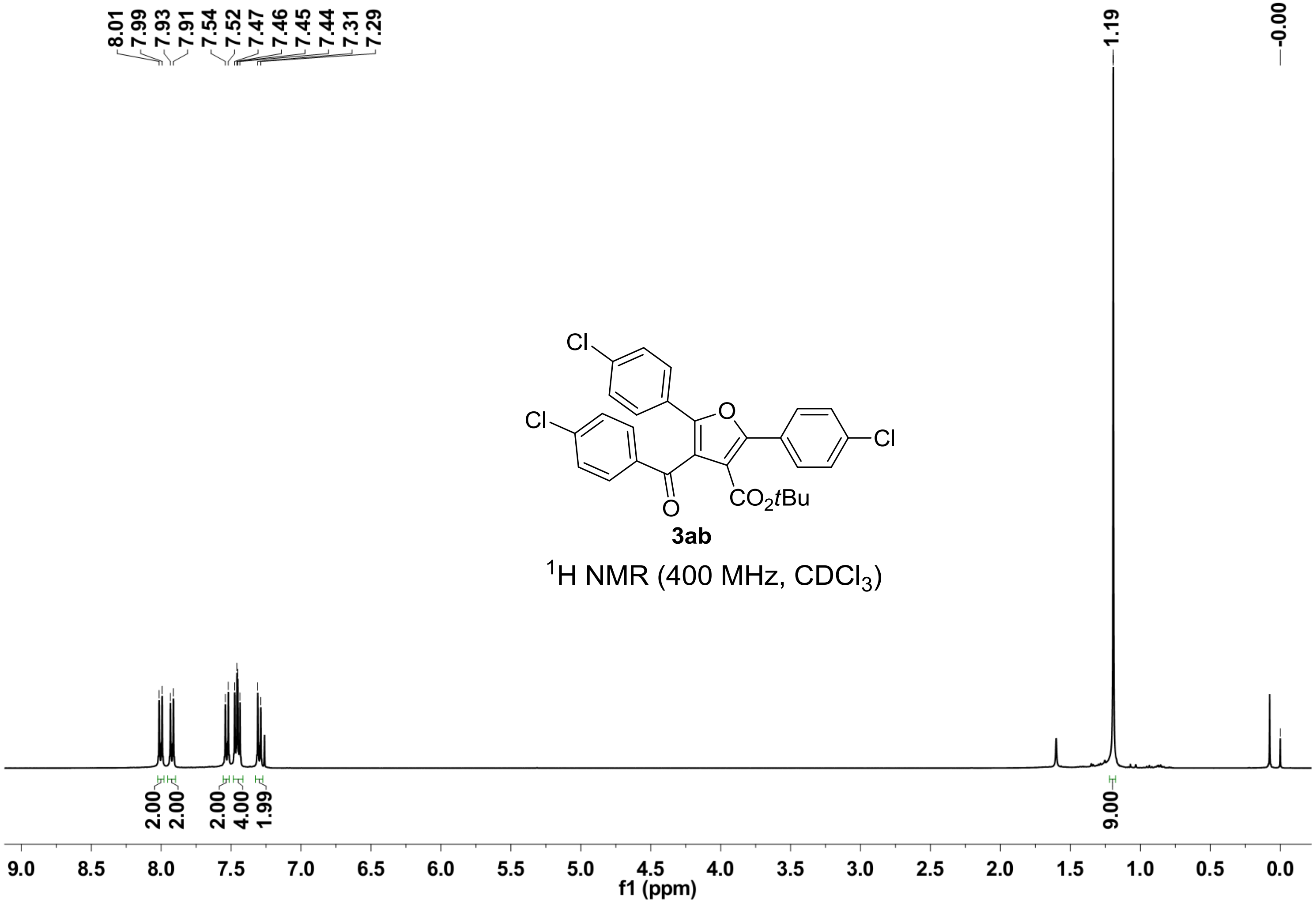

3ab

^{1}H NMR (400 MHz, $CDCl_3$)

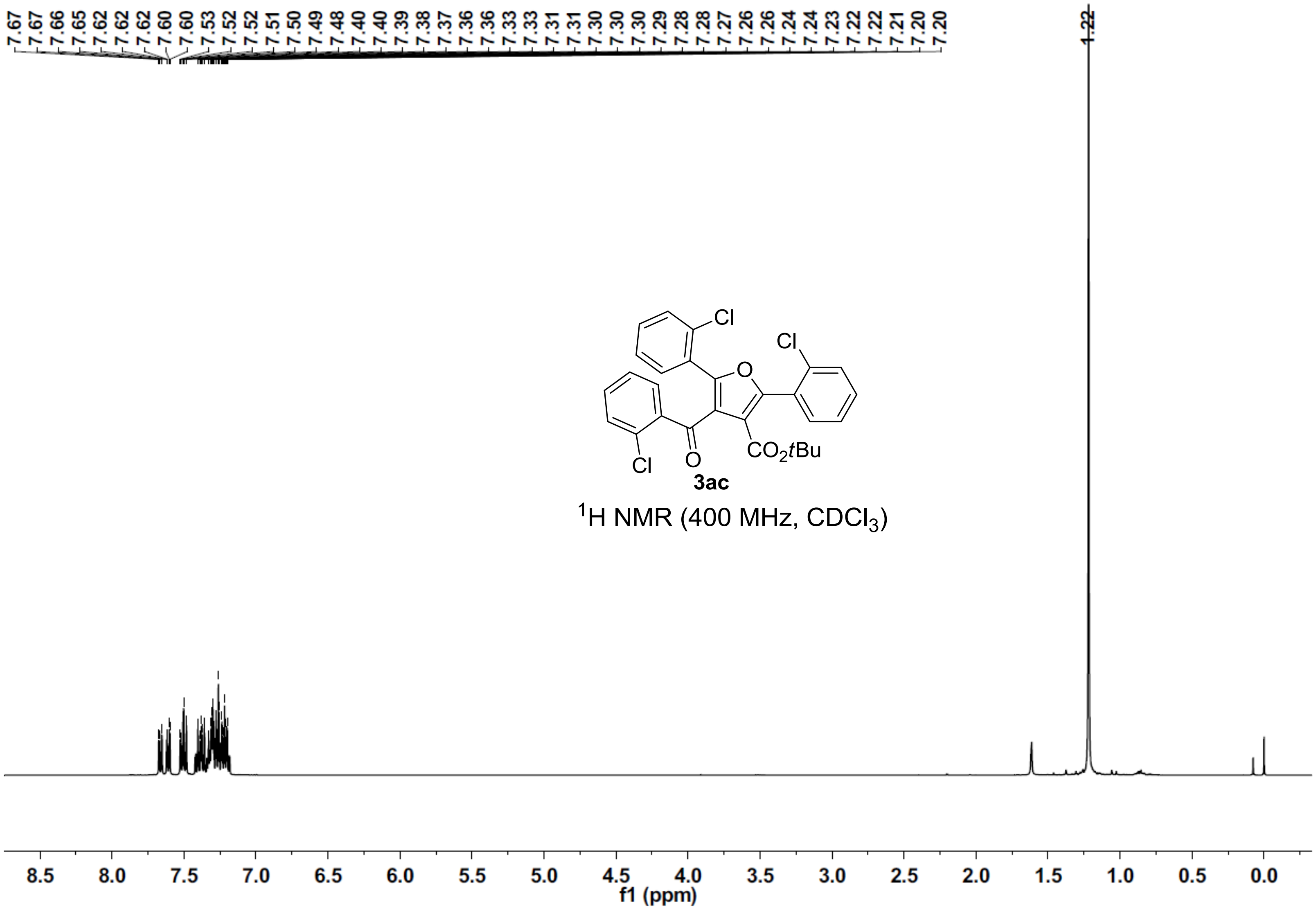
7.67
7.67
7.66
7.65
7.62
7.62
7.62
7.60
7.60
7.53
7.52
7.52
7.51
7.50
7.49
7.48
7.40
7.40
7.39
7.38
7.37
7.36
7.36
7.33
7.33
7.31
7.31
7.30
7.30
7.30
7.29
7.28
7.28
7.27
7.26
7.26
7.24
7.24
7.23
7.22
7.22
7.22
7.21
7.20
7.20
1.22
Cl
Cl
O
Cl
O
CO_2tBu
3ac
^1H NMR (400 MHz, $CDCl_3$)
8.5 8.0 7.5 7.0 6.5 6.0 5.5 5.0 4.5 4.0 3.5 3.0 2.5 2.0 1.5 1.0 0.5 0.0
f1 (ppm)

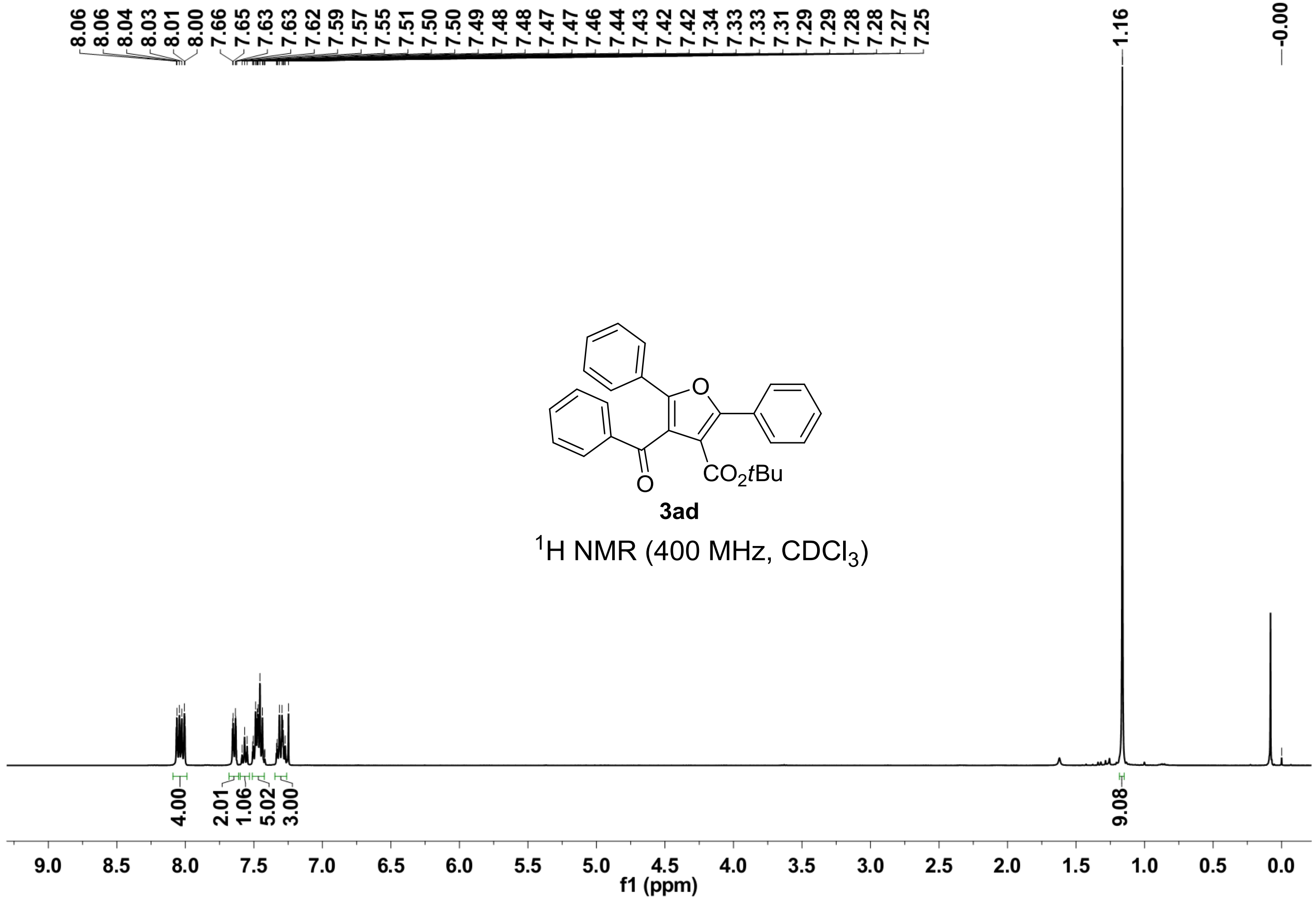
8.06
8.06
8.04
8.03
8.01
8.00
7.66
7.65
7.63
7.63
7.62
7.59
7.57
7.55
7.51
7.50
7.50
7.49
7.48
7.48
7.47
7.47
7.46
7.44
7.43
7.42
7.42
7.34
7.33
7.33
7.31
7.29
7.29
7.28
7.28
7.27
7.25
1.16
-0.00
O
O
CO2tBu
3ad
1H NMR (400 MHz, CDCl3)
4.00
2.01
1.06
5.02
3.00
9.08
9.0
8.5
8.0
7.5
7.0
6.5
6.0
5.5
5.0
4.5
4.0
3.5
3.0
2.5
2.0
1.5
1.0
0.5
0.0
f1 (ppm)

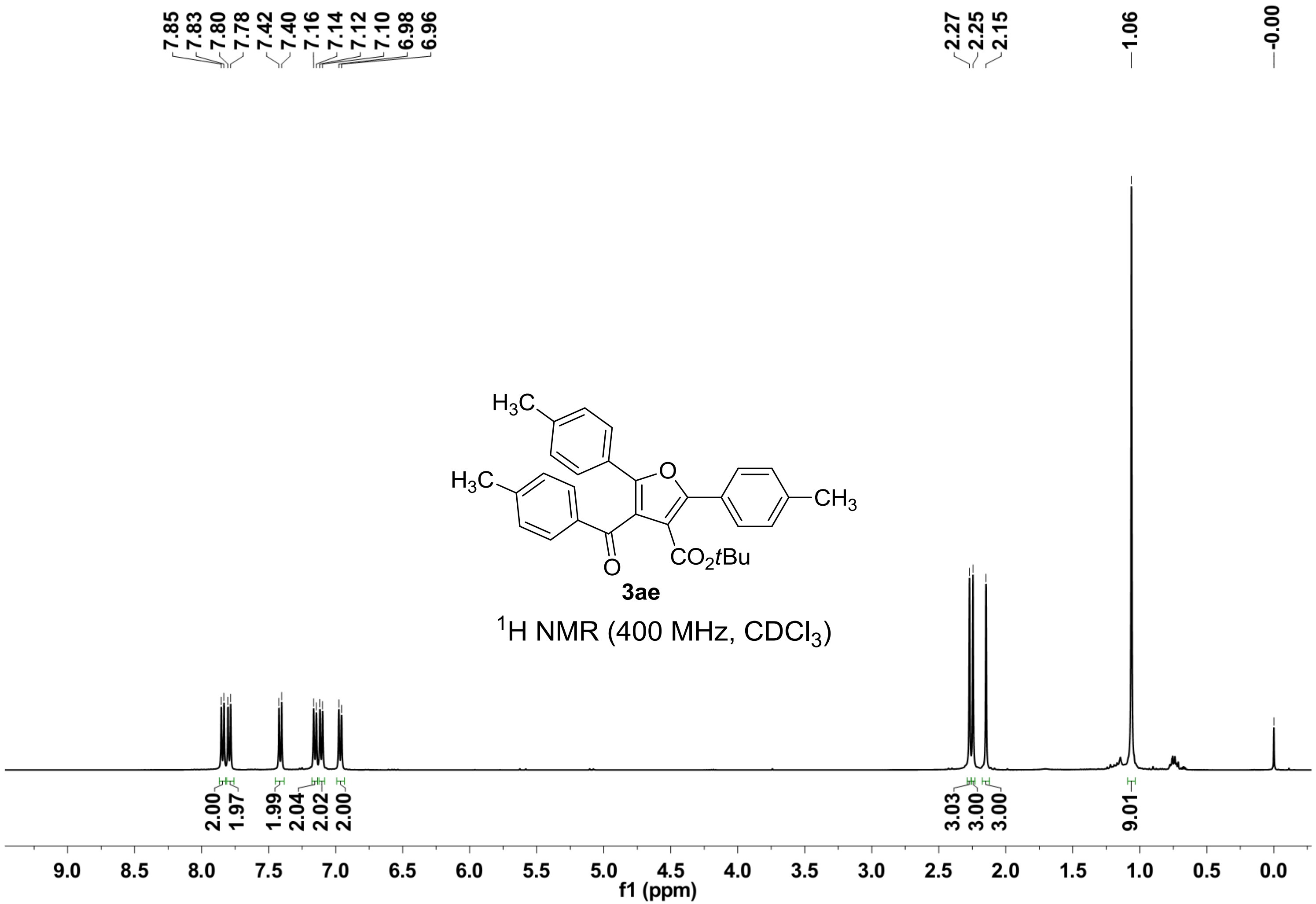
7.85
7.83
7.80
7.78
7.42
7.40
7.16
7.14
7.12
7.10
6.98
6.96
2.27
2.25
2.15
1.06
-0.00
3ae
CO2tBu
1H NMR (400 MHz, CDCl3)
2.00
1.97
1.99
2.04
2.02
2.00
3.03
3.00
3.00
9.01
f1 (ppm)

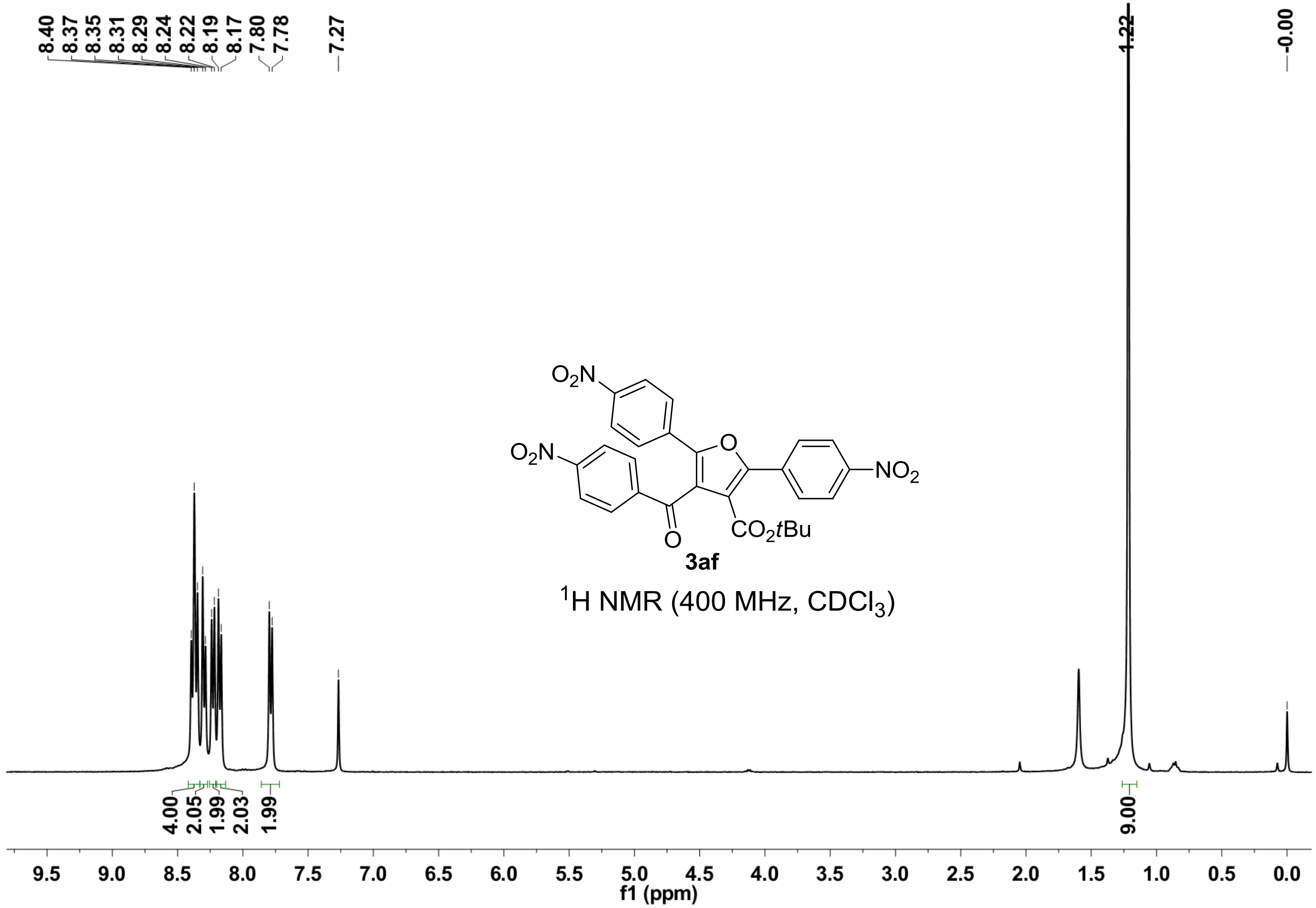
8.40
8.37
8.35
8.31
8.29
8.24
8.22
8.19
8.17
7.80
7.78
7.27
1.22
-0.00
O_2N
O_2N
O
NO_2
O
CO_2tBu
3af
^{1}H NMR (400 MHz, $CDCl_3$)
4.00
2.05
1.99
2.03
1.99
9.00
9.5
9.0
8.5
8.0
7.5
7.0
6.5
6.0
5.5
5.0
4.5
4.0
3.5
3.0
2.5
2.0
1.5
1.0
0.5
0.0
f1 (ppm)

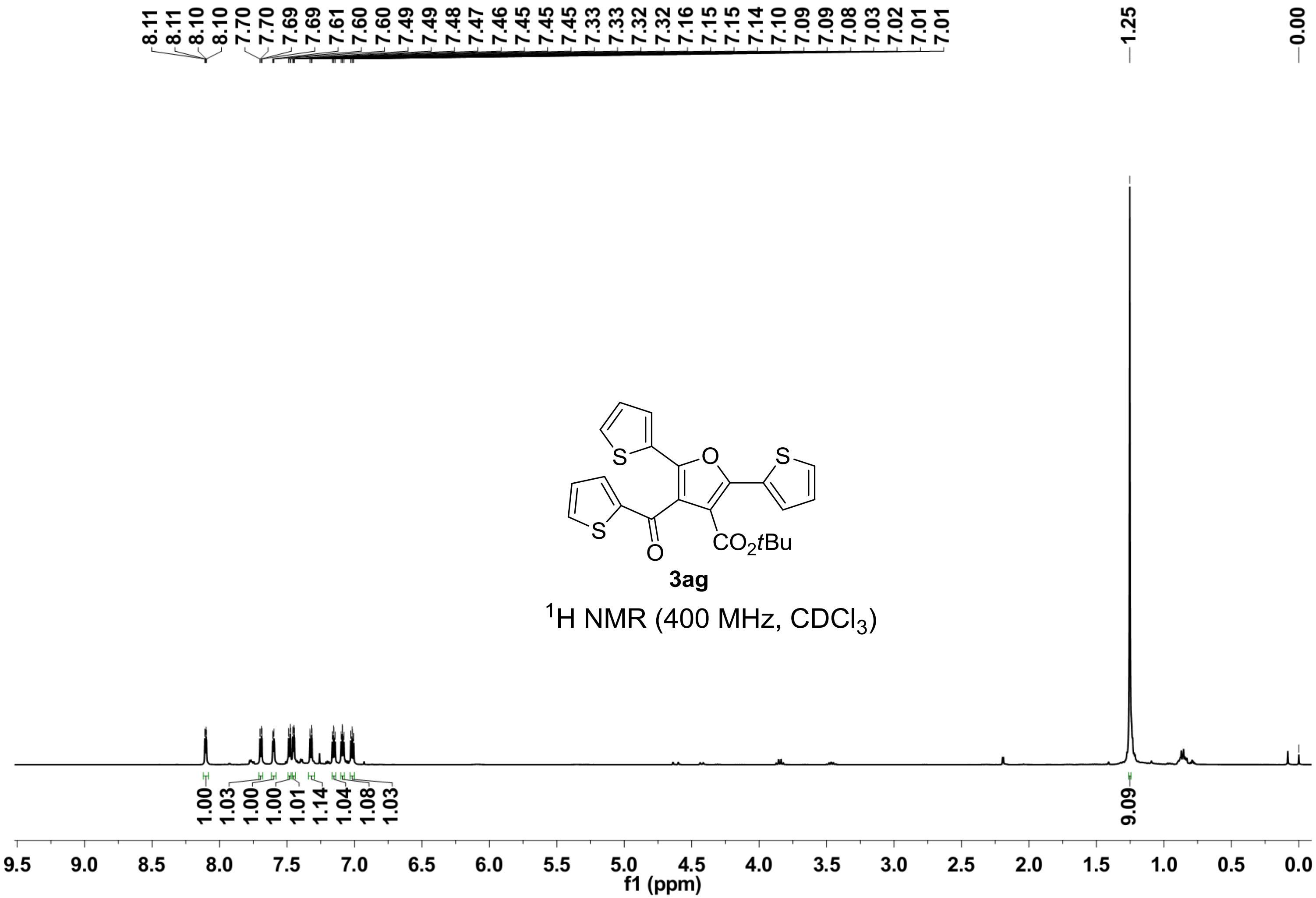
8.11 8.11 8.10 8.10 7.70 7.70 7.69 7.69 7.61 7.60 7.60 7.49 7.49 7.48 7.47 7.46 7.45 7.45 7.45 7.33 7.33 7.32 7.32 7.16 7.15 7.15 7.14 7.10 7.09 7.09 7.08 7.03 7.02 7.01 7.01
1.25
0.00
S
O
S
S
O
CO2tBu
3ag
1H NMR (400 MHz, CDCl3)
1.00 1.03 1.00 1.00 1.01 1.14 1.04 1.08 1.03
9.09
9.5 9.0 8.5 8.0 7.5 7.0 6.5 6.0 5.5 5.0 4.5 4.0 3.5 3.0 2.5 2.0 1.5 1.0 0.5 0.0
f1 (ppm)

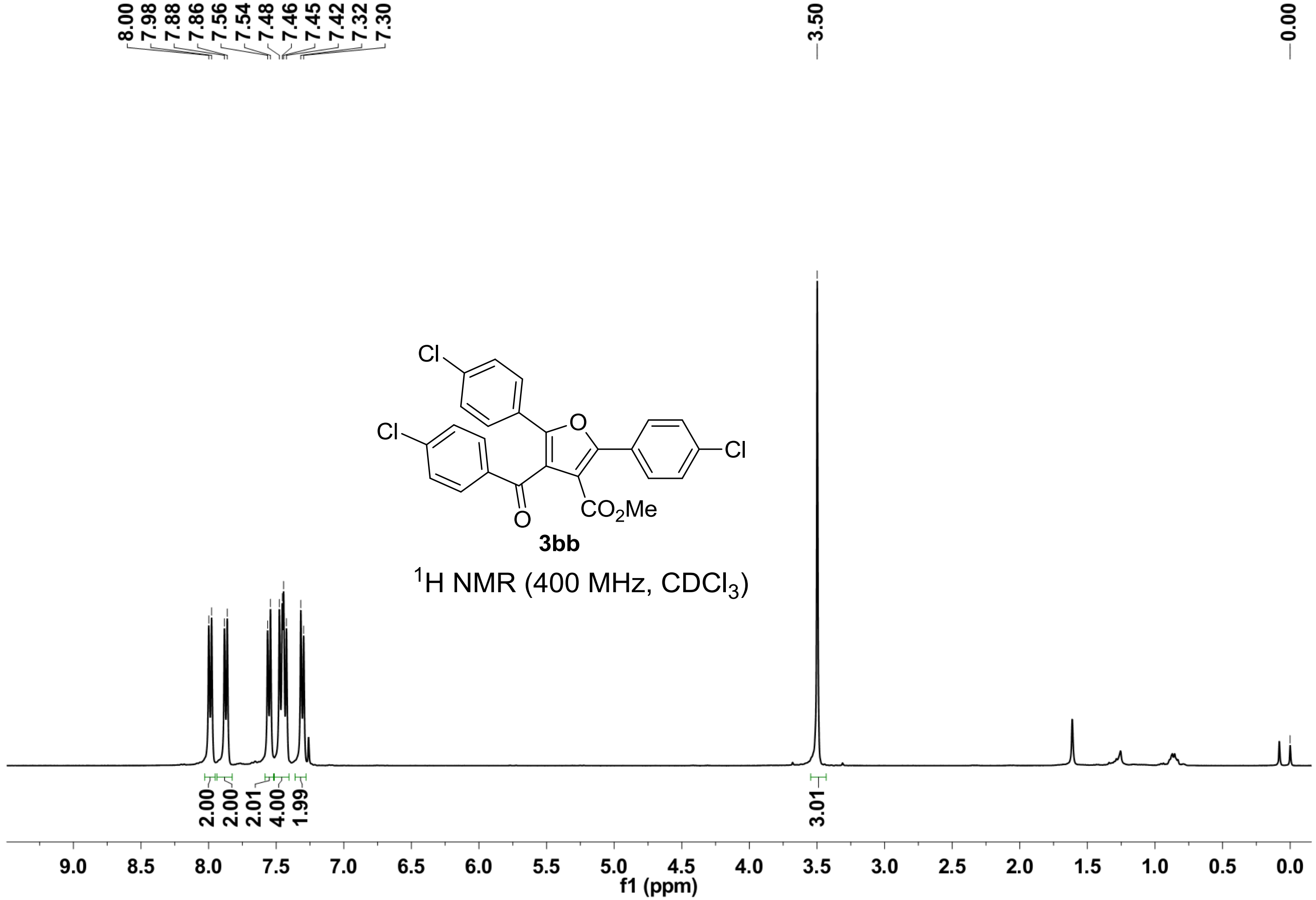

8.00
7.98
7.88
7.86
7.56
7.54
7.48
7.46
7.45
7.42
7.32
7.30
3.50
0.00
Cl
Cl
O
Cl
O
CO2Me
3bb
1H NMR (400 MHz, CDCl3)
2.00
2.00
2.01
4.00
1.99
3.01
9.0
8.5
8.0
7.5
7.0
6.5
6.0
5.5
5.0
4.5
4.0
3.5
3.0
2.5
2.0
1.5
1.0
0.5
0.0
f1 (ppm)

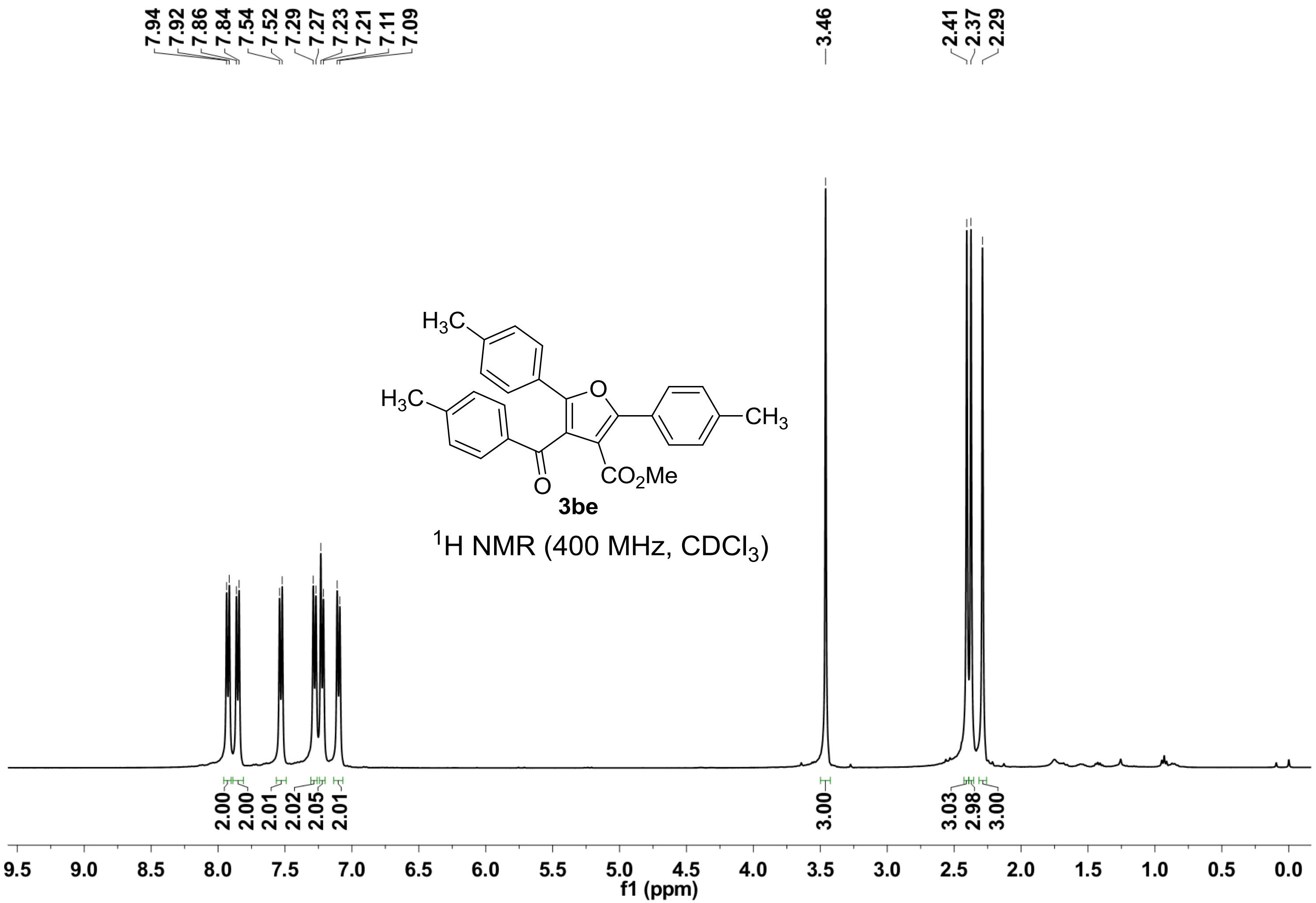
7.94
7.92
7.86
7.84
7.54
7.52
7.29
7.27
7.23
7.21
7.11
7.09
3.46
2.41
2.37
2.29
H3C
H3C
O
CH3
O
CO2Me
3be
1H NMR (400 MHz, CDCl3)
2.00
2.00
2.01
2.02
2.05
2.01
3.00
3.03
2.98
3.00
9.5
9.0
8.5
8.0
7.5
7.0
6.5
6.0
5.5
5.0
4.5
4.0
3.5
3.0
2.5
2.0
1.5
1.0
0.5
0.0
f1 (ppm)

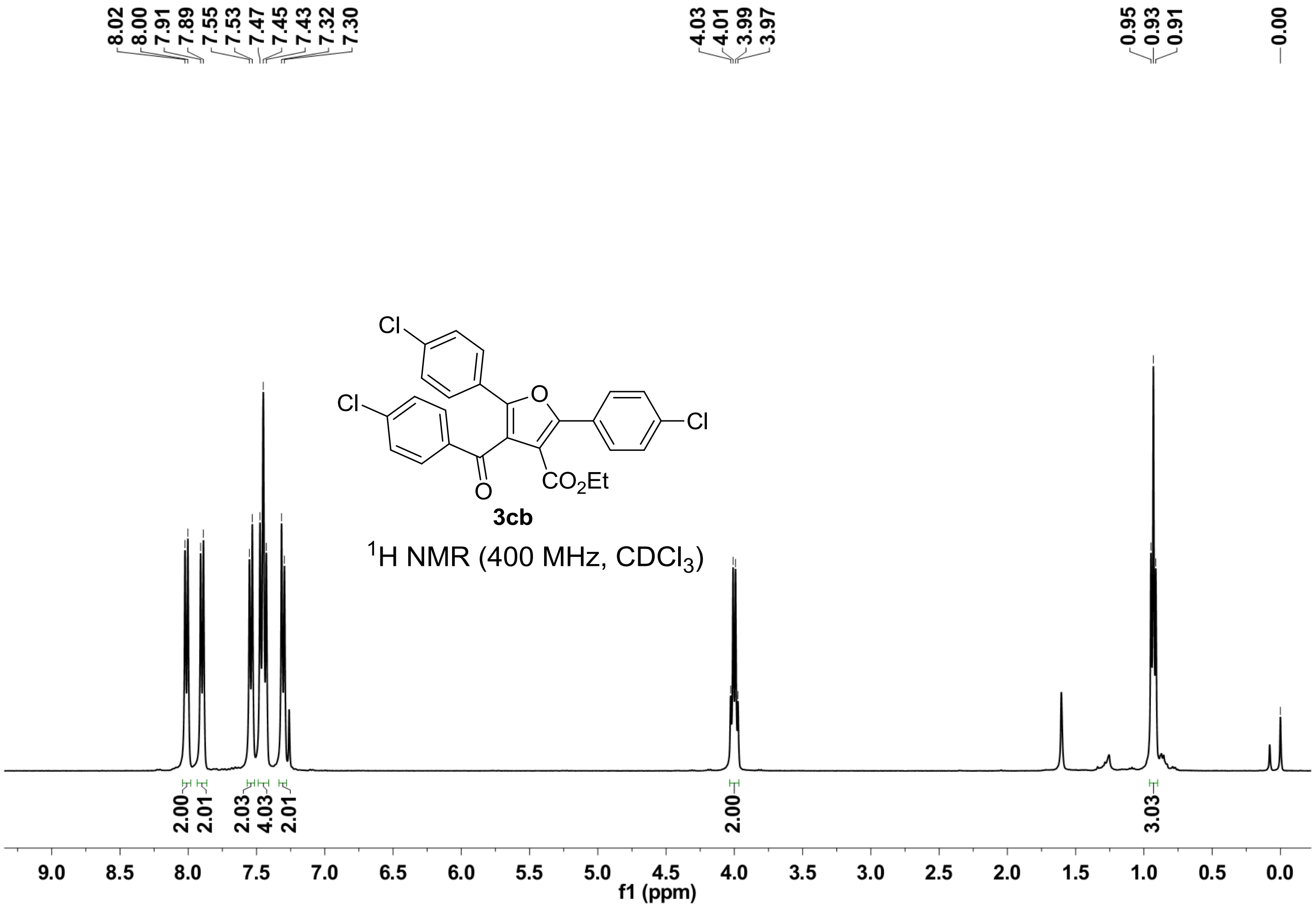
8.02
8.00
7.91
7.89
7.55
7.53
7.47
7.45
7.43
7.32
7.30
4.03
4.01
3.99
3.97
0.95
0.93
0.91
0.00
Cl
Cl
O
Cl
O
CO2Et
3cb
1H NMR (400 MHz, CDCl3)
2.00
2.01
2.03
4.03
2.01
2.00
3.03
9.0
8.5
8.0
7.5
7.0
6.5
6.0
5.5
5.0
4.5
4.0
3.5
3.0
2.5
2.0
1.5
1.0
0.5
0.0
f1 (ppm)

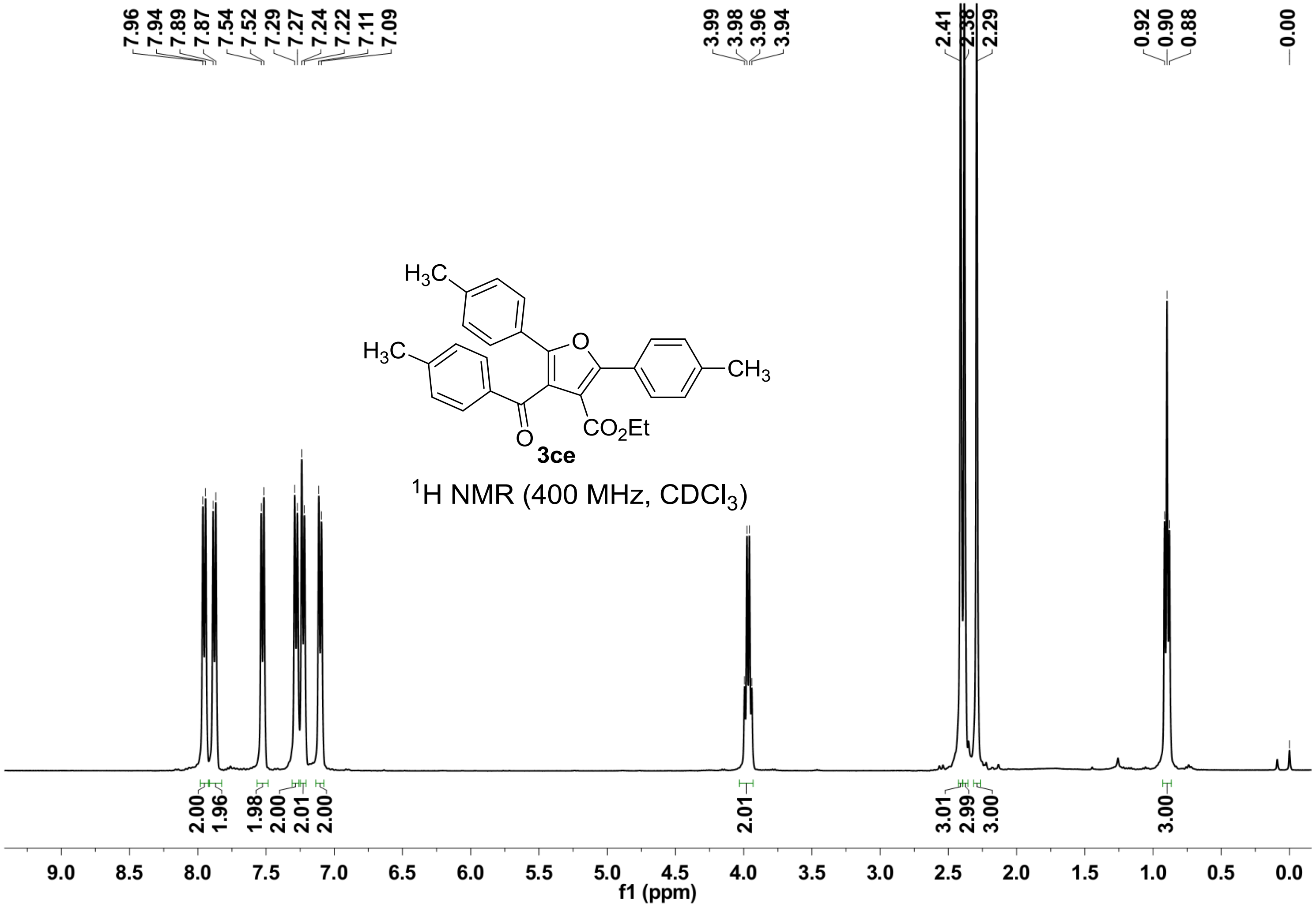
7.96 7.94 7.89 7.87 7.54 7.52 7.29 7.27 7.24 7.22 7.11 7.09
3.99 3.98 3.96 3.94
2.41 2.38 2.29
0.92 0.90 0.88
0.00
3ce
^{1}H NMR (400 MHz, $CDCl_3$)
2.00 1.96 1.98 2.00 2.01 2.00 2.01 3.01 2.99 3.00 3.00
9.0 8.5 8.0 7.5 7.0 6.5 6.0 5.5 5.0 4.5 4.0 3.5 3.0 2.5 2.0 1.5 1.0 0.5 0.0
f1 (ppm)

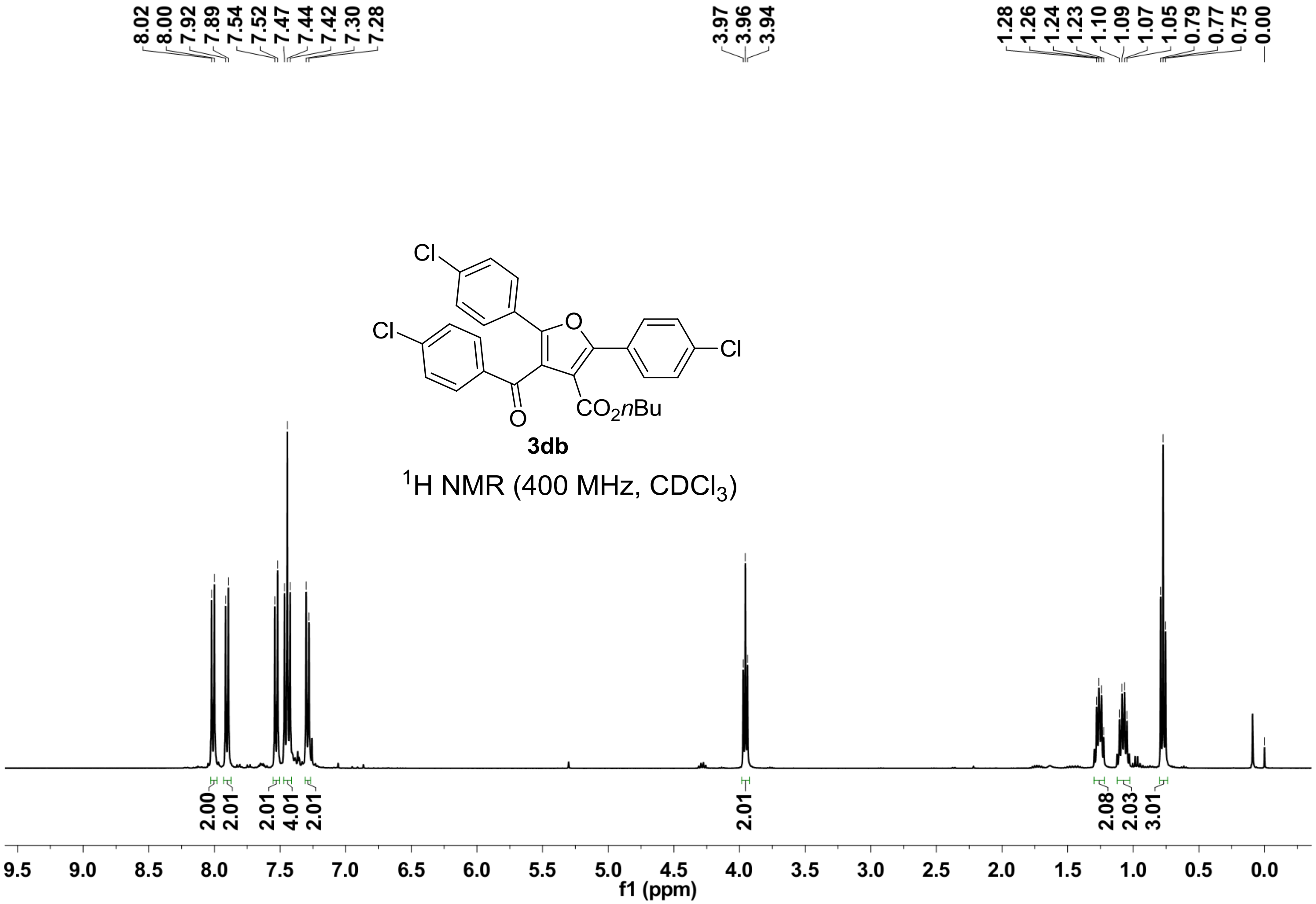
8.02
8.00
7.92
7.89
7.54
7.52
7.47
7.44
7.42
7.30
7.28
3.97
3.96
3.94
1.28
1.26
1.24
1.23
1.10
1.09
1.07
1.05
0.79
0.77
0.75
0.00
Cl
O
CO2nBu
3db
1H NMR (400 MHz, CDCl3)
2.00
2.01
2.01
4.01
2.01
2.01
2.08
2.03
3.01
9.5 9.0 8.5 8.0 7.5 7.0 6.5 6.0 5.5 5.0 4.5 4.0 3.5 3.0 2.5 2.0 1.5 1.0 0.5 0.0
f1 (ppm)

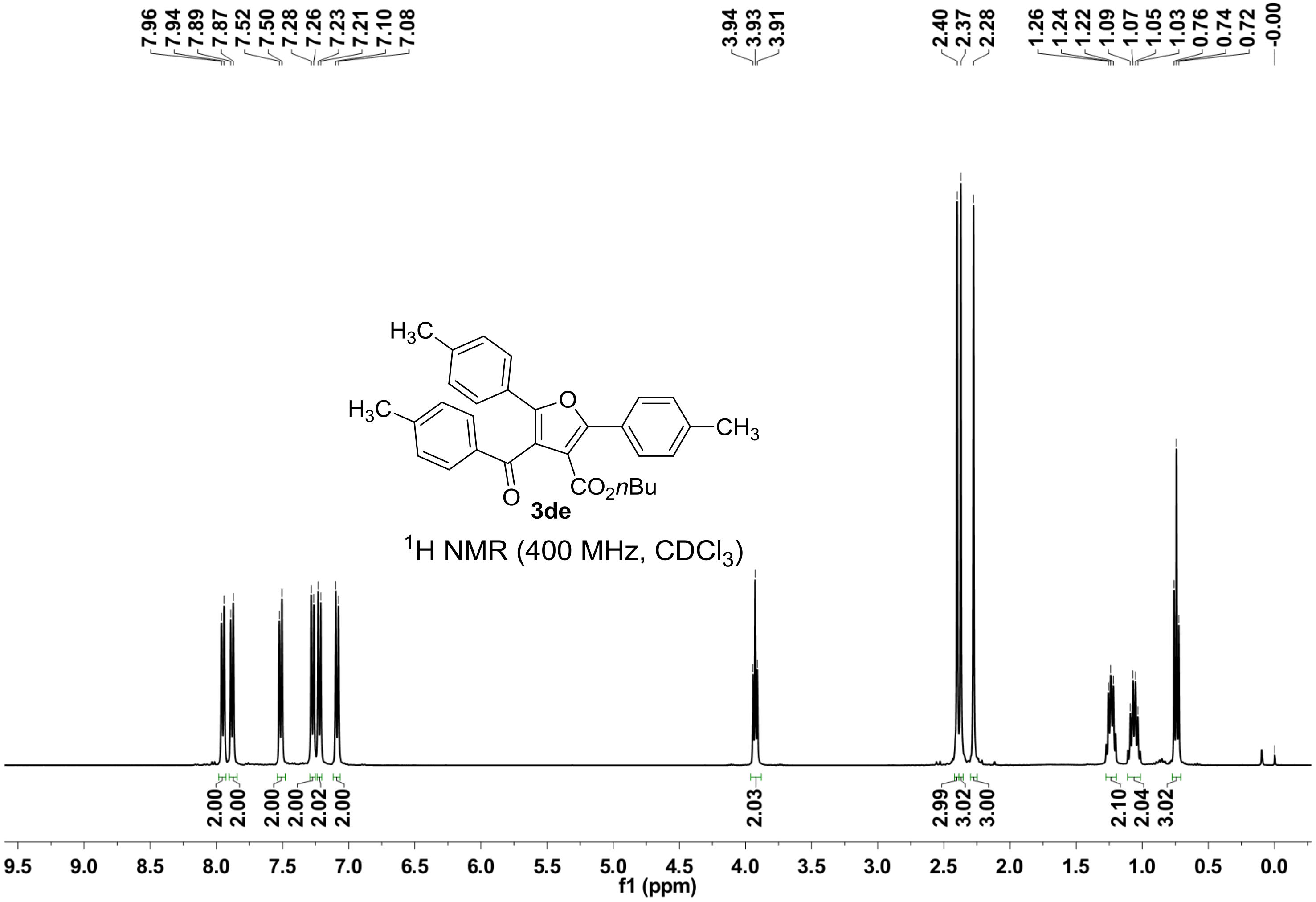

7.96
7.94
7.89
7.87
7.52
7.50
7.28
7.26
7.23
7.21
7.10
7.08
3.94
3.93
3.91
2.40
2.37
2.28
1.26
1.24
1.22
1.09
1.07
1.05
1.03
0.76
0.74
0.72
-0.00
H3C
H3C
O
CH3
O
CO2nBu
3de
1H NMR (400 MHz, CDCl3)
2.00
2.00
2.00
2.00
2.02
2.00
2.03
2.99
3.02
3.00
2.10
2.04
3.02
9.5
9.0
8.5
8.0
7.5
7.0
6.5
6.0
5.5
5.0
4.5
4.0
3.5
3.0
2.5
2.0
1.5
1.0
0.5
0.0
f1 (ppm)

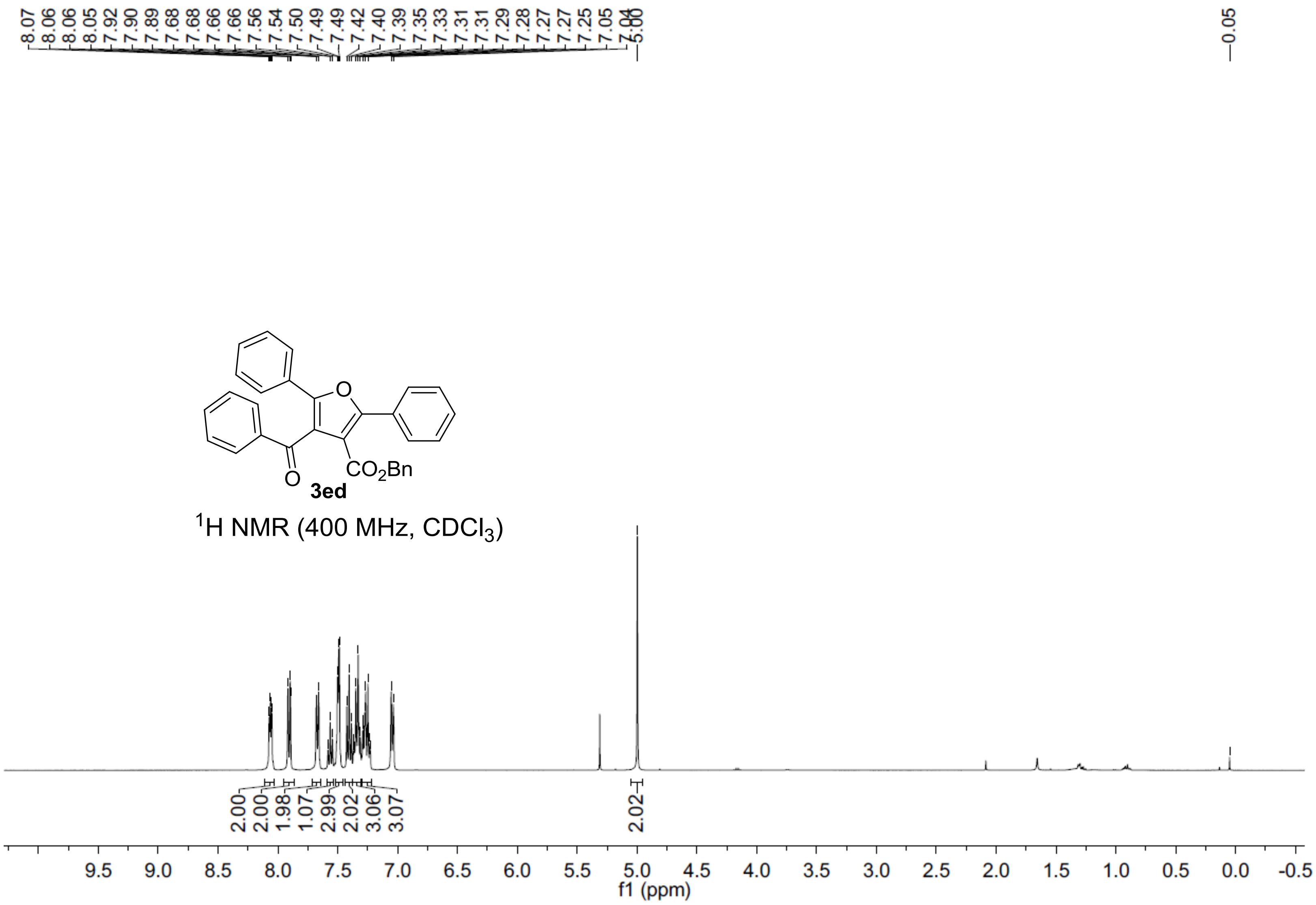
3ed

^{1}H NMR (400 MHz, $CDCl_3$)

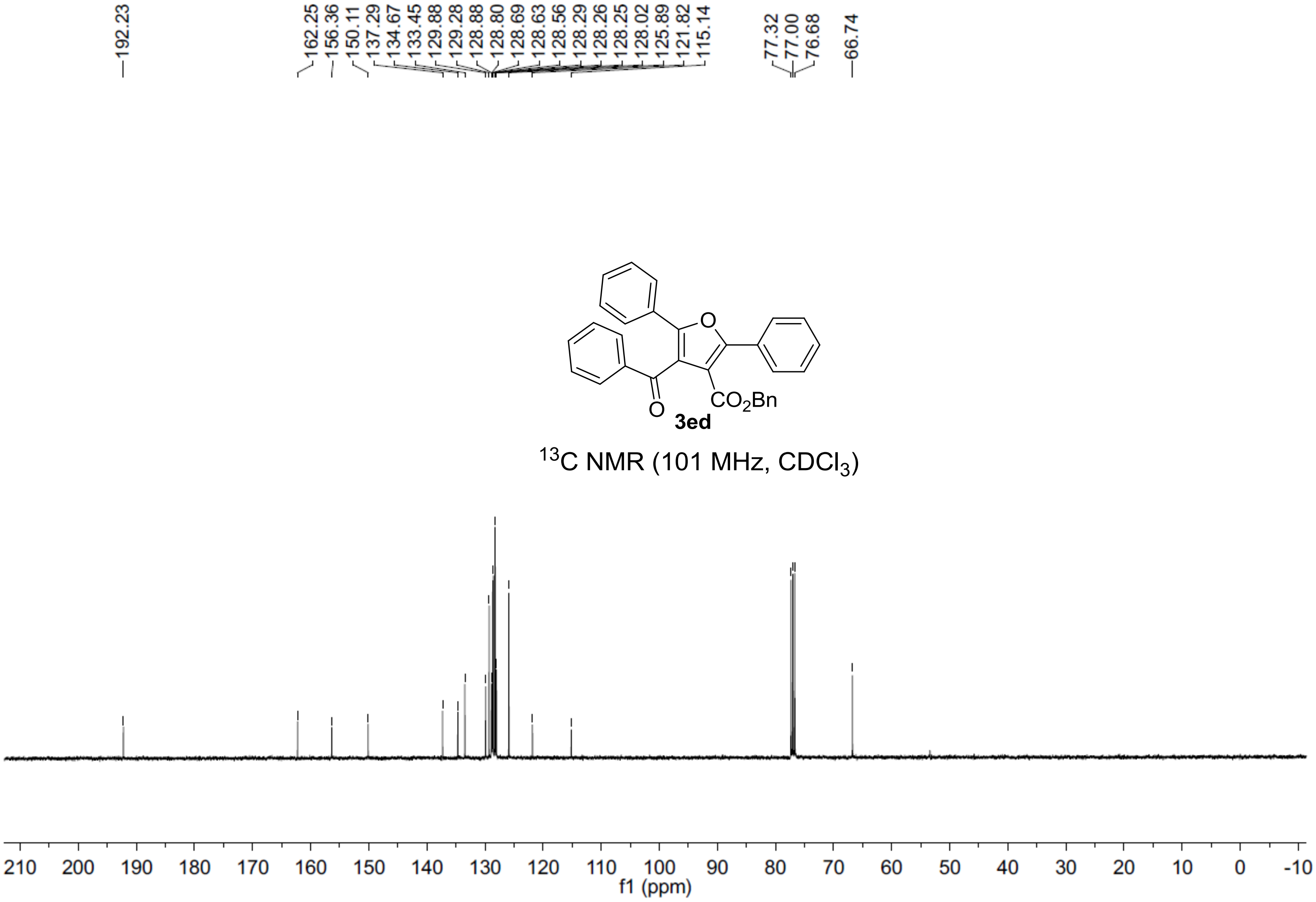

192.23
162.25
156.36
150.11
137.29
134.67
133.45
129.88
129.28
128.88
128.80
128.69
128.63
128.56
128.29
128.26
128.25
128.02
125.89
121.82
115.14
77.32
77.00
76.68
66.74
O
CO2Bn
3ed
13C NMR (101 MHz, CDCl3)
210
200
190
180
170
160
150
140
130
120
110
100
90
80
70
60
50
40
30
20
10
0
-10
f1 (ppm)

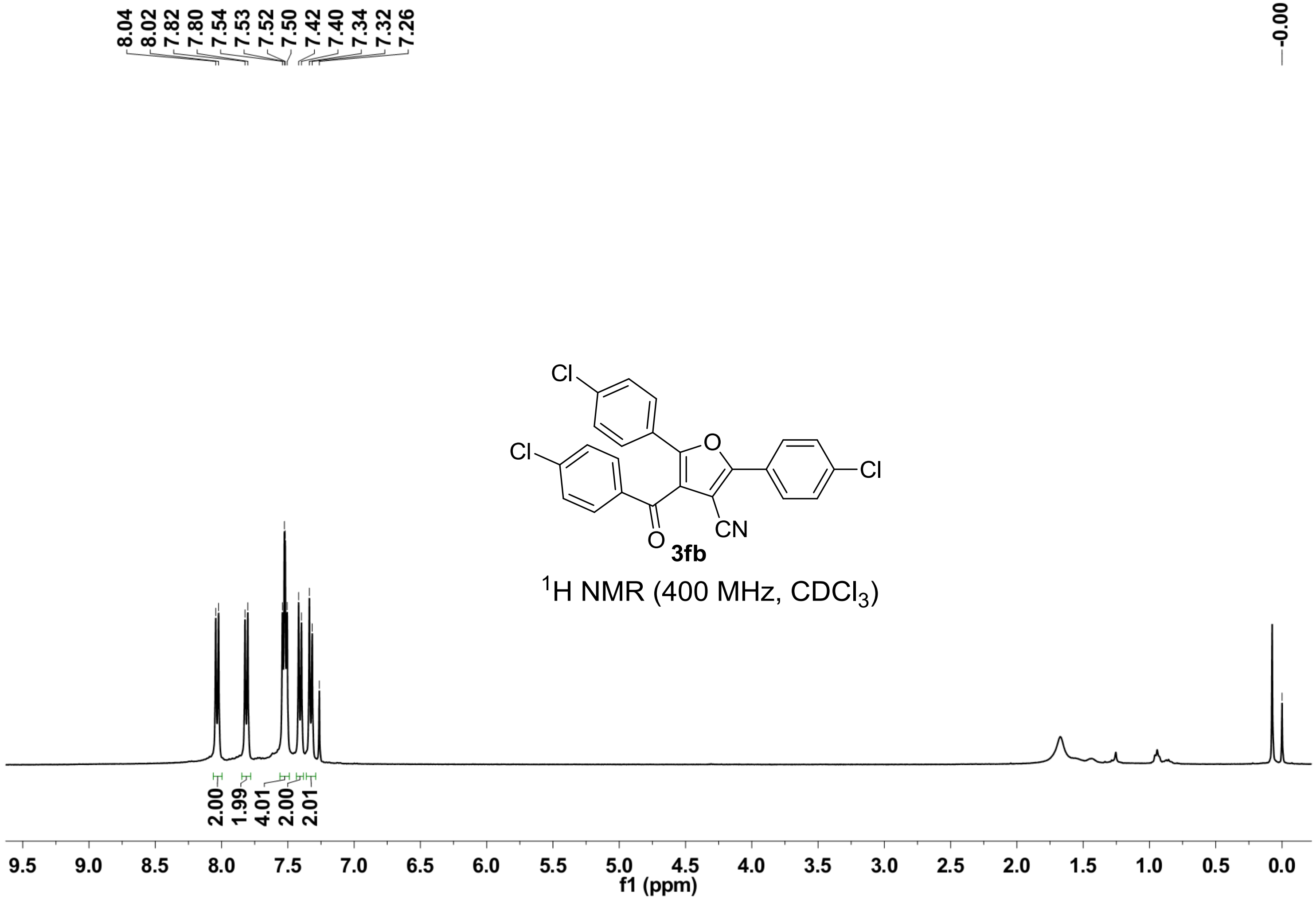
8.04
8.02
7.82
7.80
7.54
7.53
7.52
7.50
7.42
7.40
7.34
7.32
7.26
-0.00
Cl
Cl
O
Cl
O
CN
3fb
1H NMR (400 MHz, CDCl3)
2.00
1.99
4.01
2.00
2.01
9.5
9.0
8.5
8.0
7.5
7.0
6.5
6.0
5.5
5.0
4.5
4.0
3.5
3.0
2.5
2.0
1.5
1.0
0.5
0.0
f1 (ppm)

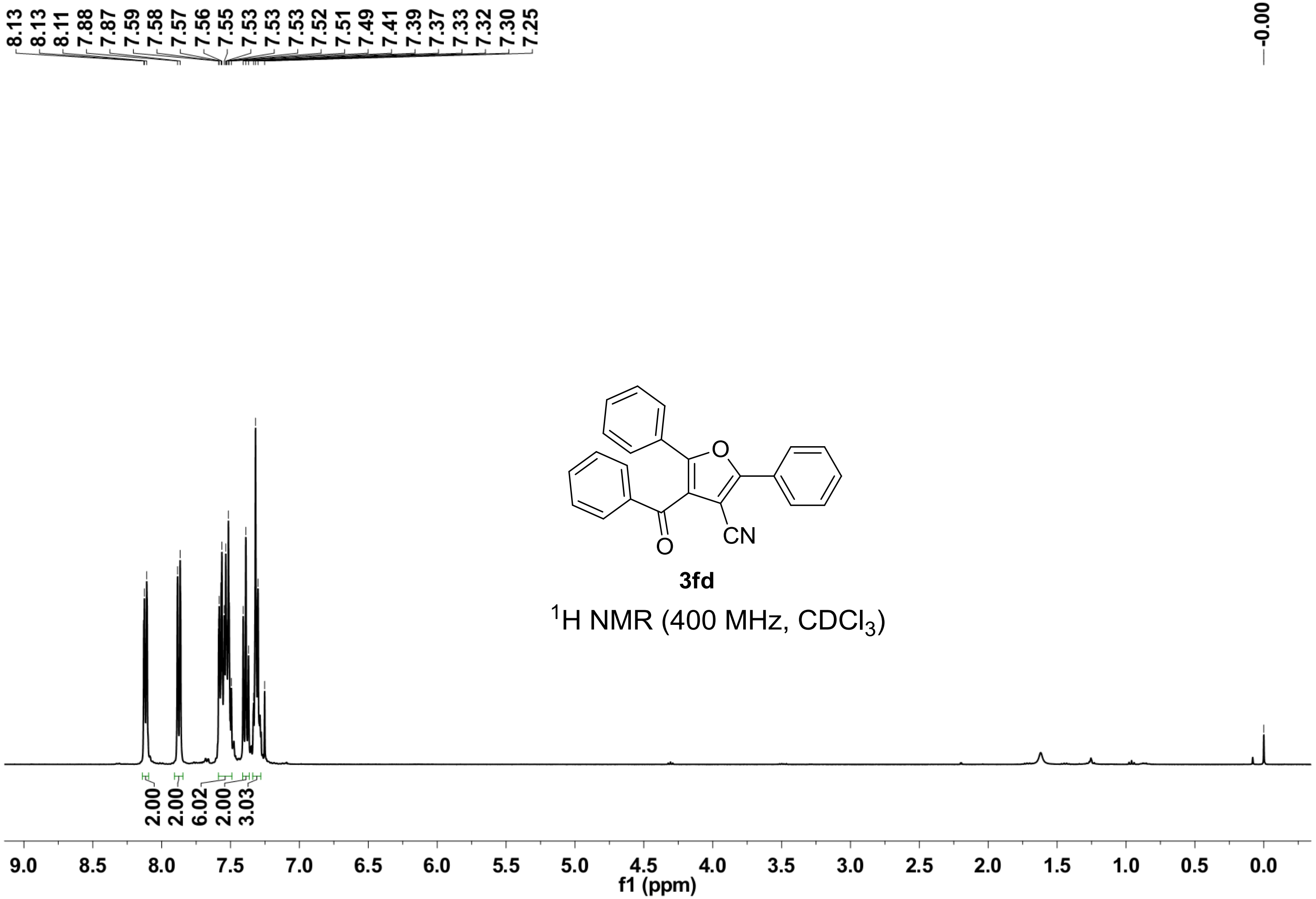

3fd

^{1}H NMR (400 MHz, $CDCl_3$)

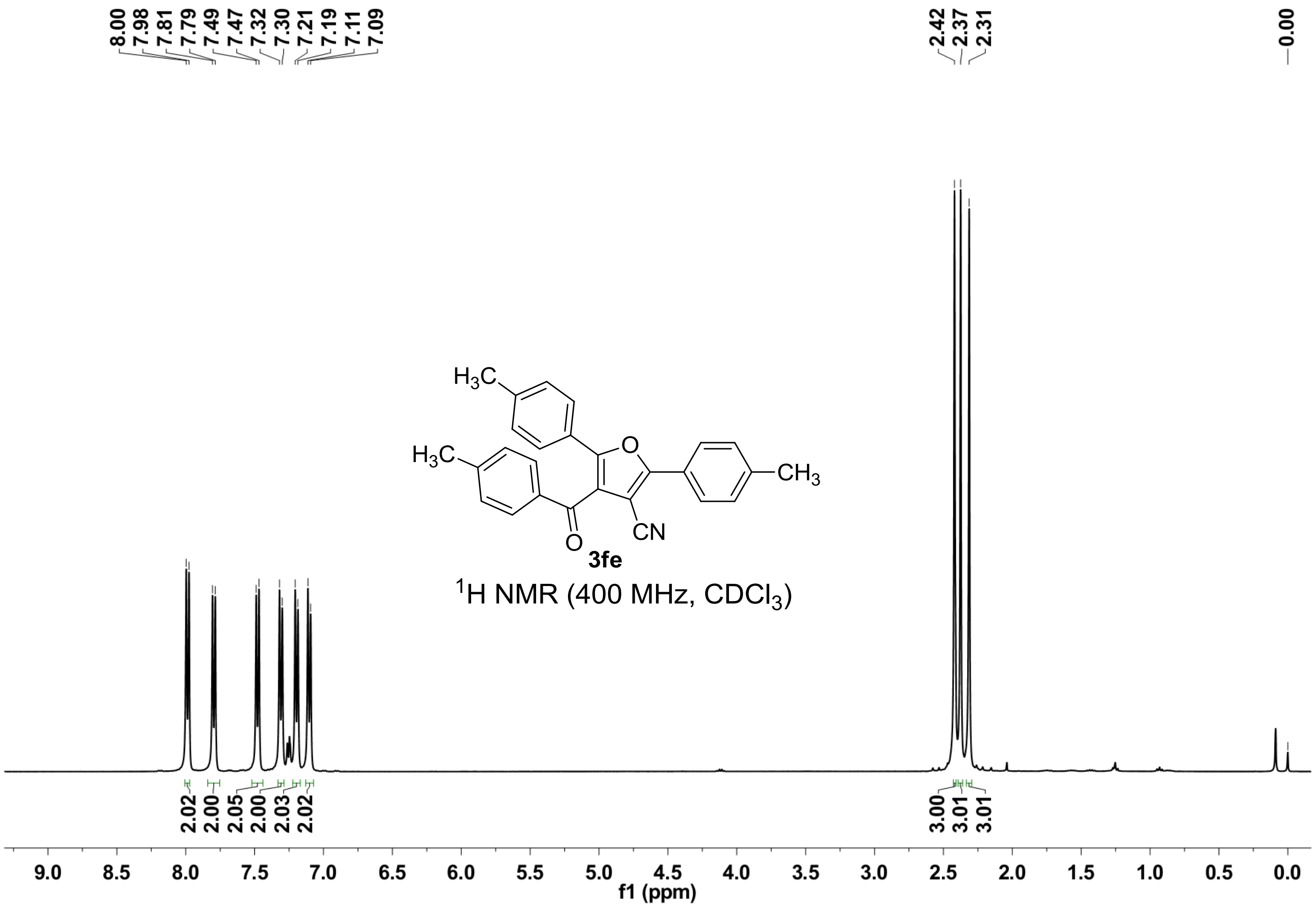

8.00
7.98
7.81
7.79
7.49
7.47
7.32
7.30
7.21
7.19
7.11
7.09
2.42
2.37
2.31
0.00
H_3C
H_3C
O
CH_3
CN
O
3fe
^1H NMR (400 MHz, $CDCl_3$)
2.02
2.00
2.05
2.00
2.03
2.02
3.00
3.01
3.01
9.0
8.5
8.0
7.5
7.0
6.5
6.0
5.5
5.0
4.5
4.0
3.5
3.0
2.5
2.0
1.5
1.0
0.5
0.0
f1 (ppm)

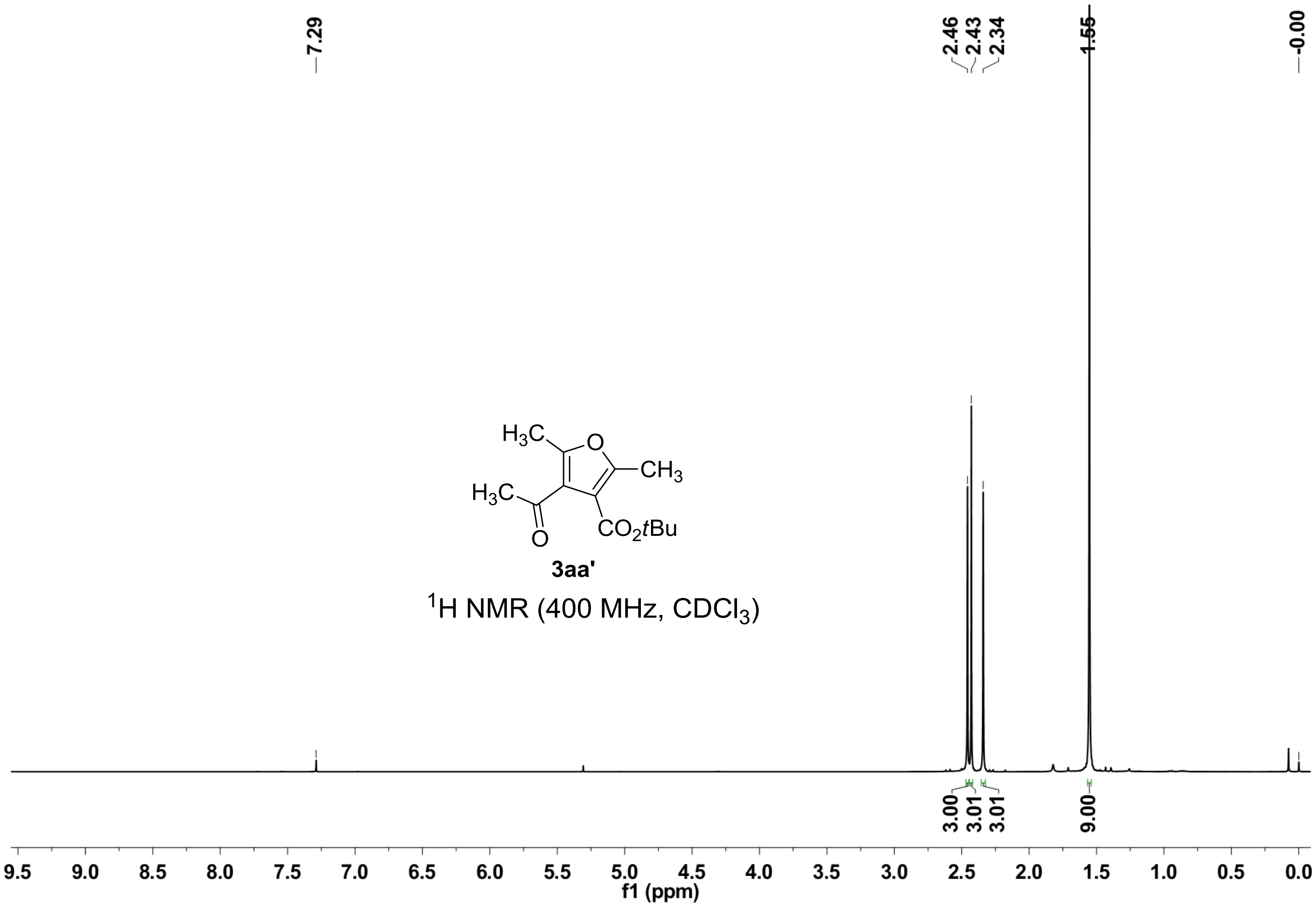
7.29
2.46
2.43
2.34
1.55
-0.00
H3C
O
CH3
H3C
O
CO2tBu
3aa'
1H NMR (400 MHz, CDCl3)
3.00
3.01
3.01
9.00
9.5 9.0 8.5 8.0 7.5 7.0 6.5 6.0 5.5 5.0 4.5 4.0 3.5 3.0 2.5 2.0 1.5 1.0 0.5 0.0
f1 (ppm)

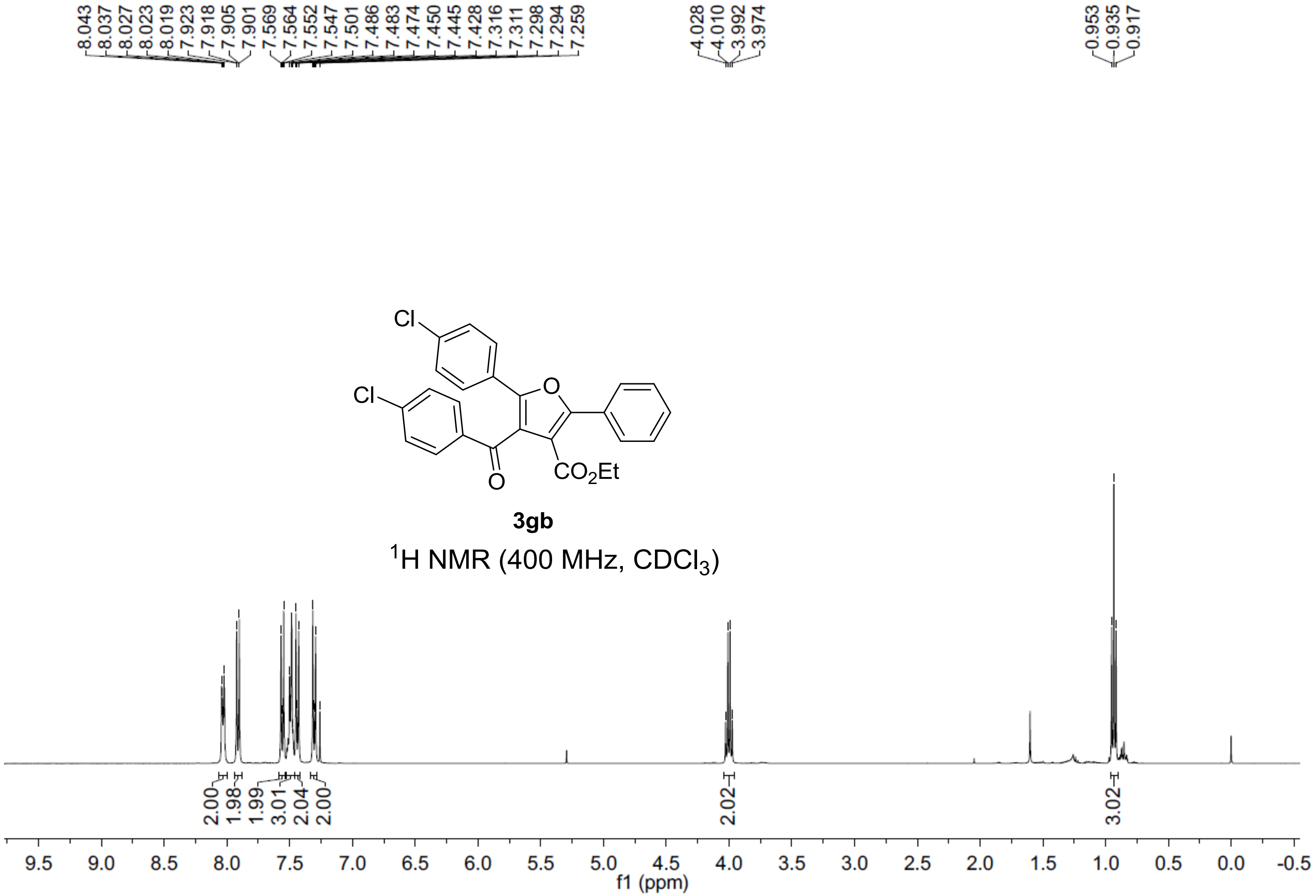

3gb

^{1}H NMR (400 MHz, $CDCl_3$)

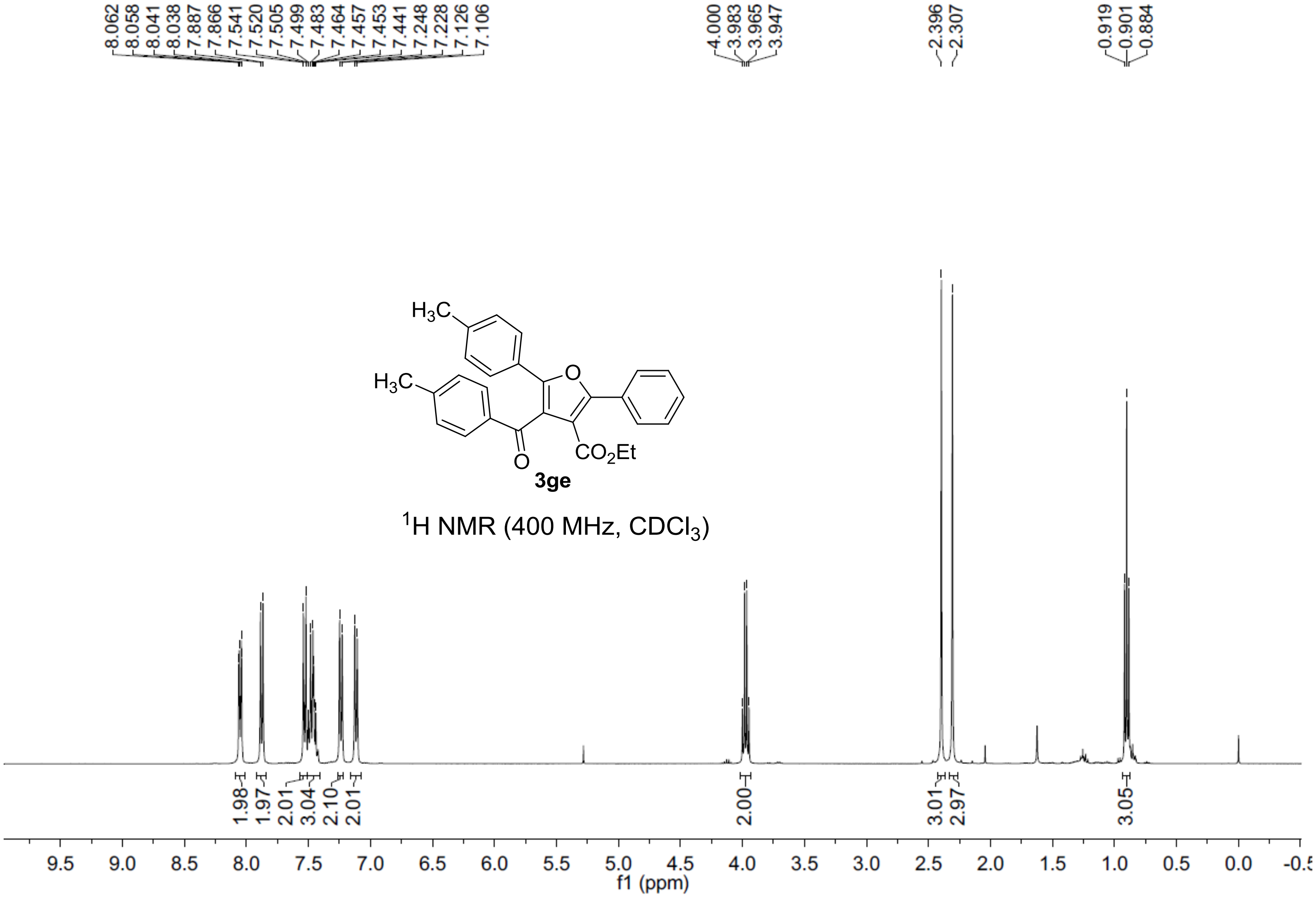
8.062
8.058
8.041
8.038
7.887
7.866
7.541
7.520
7.505
7.499
7.483
7.464
7.457
7.453
7.441
7.248
7.228
7.126
7.106
4.000
3.983
3.965
3.947
2.396
2.307
0.919
0.901
0.884
H3C
H3C
O
O
CO2Et
3ge
1H NMR (400 MHz, CDCl3)
1.98
1.97
2.01
3.04
2.10
2.01
2.00
3.01
2.97
3.05
9.5
9.0
8.5
8.0
7.5
7.0
6.5
6.0
5.5
5.0
4.5
4.0
3.5
3.0
2.5
2.0
1.5
1.0
0.5
0.0
f1 (ppm)

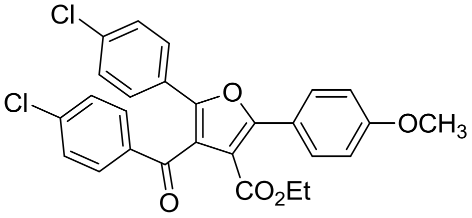

3hb

^{1}H NMR (400 MHz, $CDCl_3$)

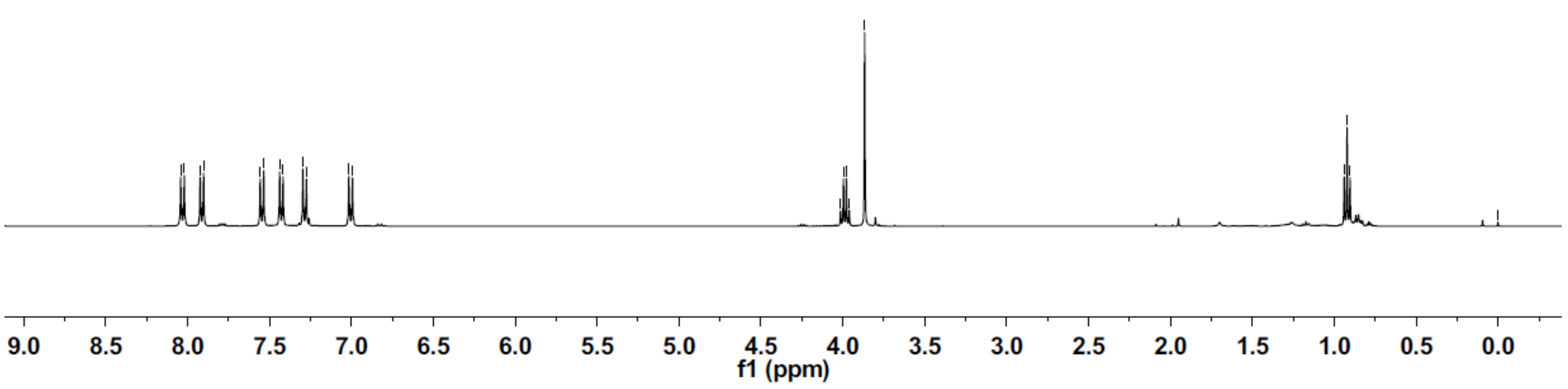

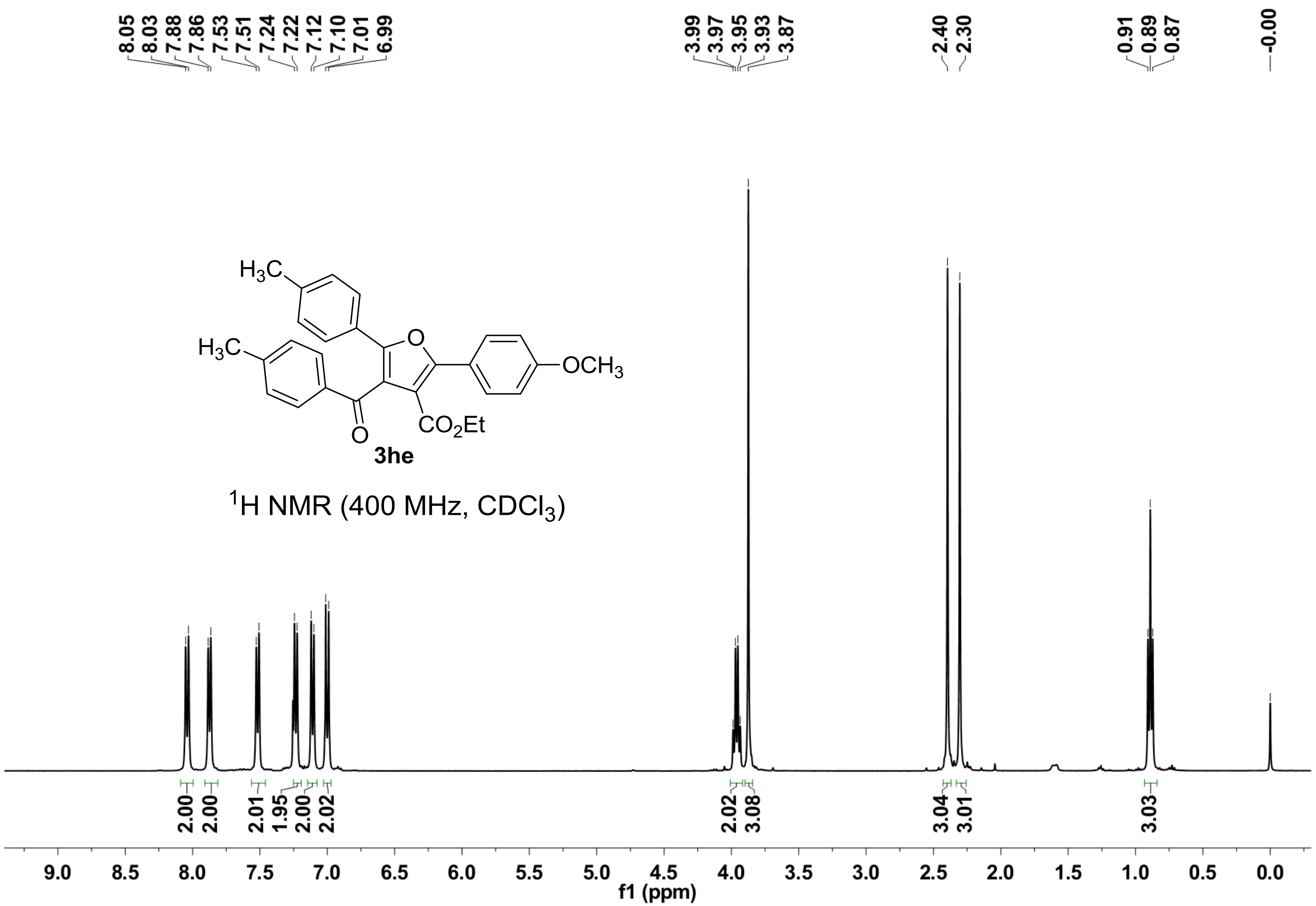

8.05
8.03
7.88
7.86
7.53
7.51
7.24
7.22
7.12
7.10
7.01
6.99
3.99
3.97
3.95
3.93
3.87
2.40
2.30
0.91
0.89
0.87
-0.00
H_3C
H_3C
O
OCH_3
O
CO_2Et
3he
^{1}H NMR (400 MHz, $CDCl_3$)
2.00
2.00
2.01
1.95
2.00
2.02
2.02
3.08
3.04
3.01
3.03
9.0
8.5
8.0
7.5
7.0
6.5
6.0
5.5
5.0
4.5
4.0
3.5
3.0
2.5
2.0
1.5
1.0
0.5
0.0
f1 (ppm)

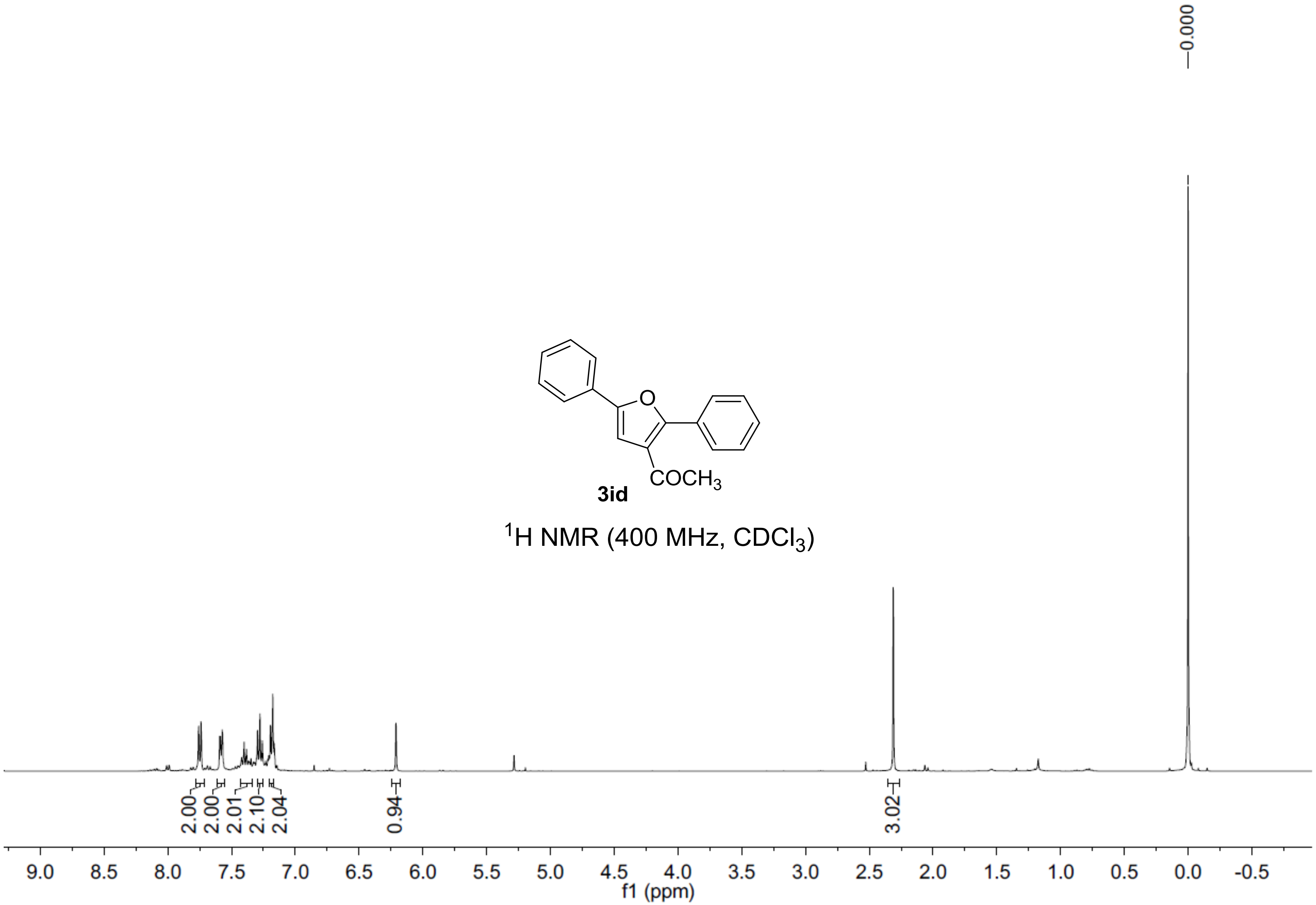
0.000
3id
COCH3
1H NMR (400 MHz, CDCl3)
2.00
2.00
2.01
2.10
2.04
0.94
3.02
9.0 8.5 8.0 7.5 7.0 6.5 6.0 5.5 5.0 4.5 4.0 3.5 3.0 2.5 2.0 1.5 1.0 0.5 0.0 -0.5
f1 (ppm)

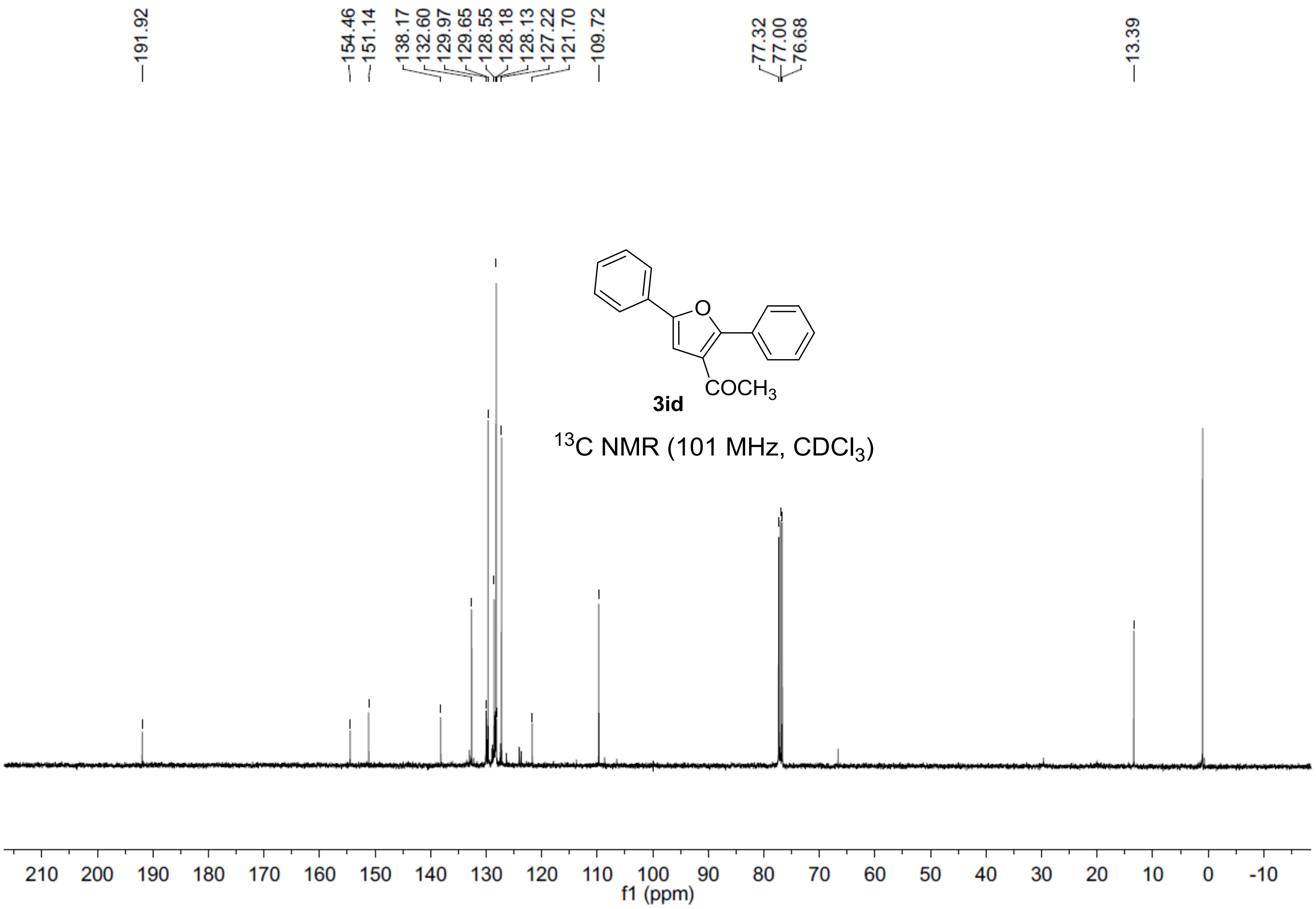
191.92
154.46
151.14
138.17
132.60
129.97
129.65
128.55
128.18
128.13
127.22
121.70
109.72
77.32
77.00
76.68
13.39
O
COCH3
3id
13C NMR (101 MHz, CDCl3)
210 200 190 180 170 160 150 140 130 120 110 100 90 80 70 60 50 40 30 20 10 0 -10
f1 (ppm)

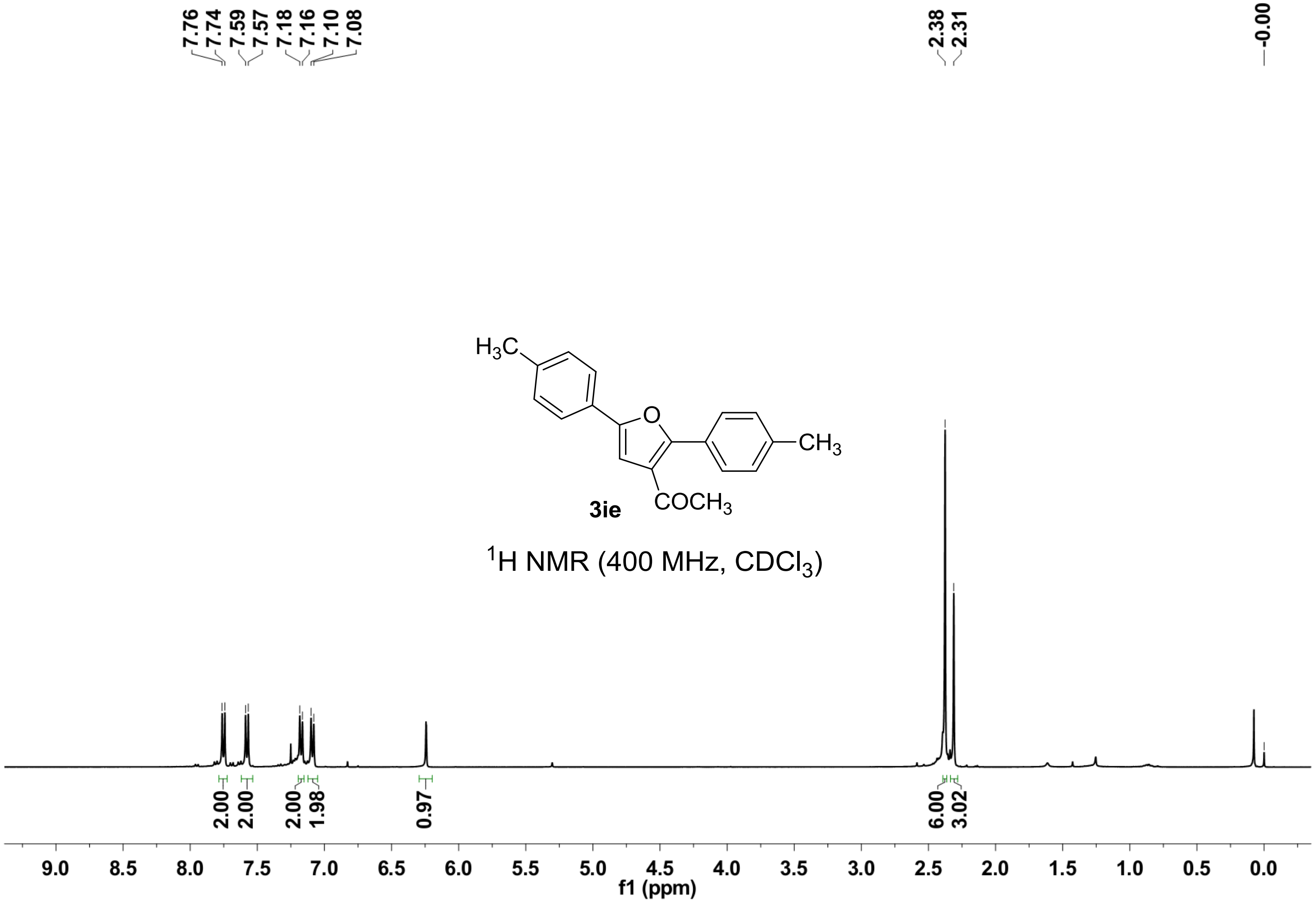

7.76
7.74
7.59
7.57
7.18
7.16
7.10
7.08
2.38
2.31
-0.00
H_3C
O
CH_3
$COCH_3$
3ie
^1H NMR (400 MHz, $CDCl_3$)
2.00
2.00
2.00
1.98
0.97
6.00
3.02
9.0
8.5
8.0
7.5
7.0
6.5
6.0
5.5
5.0
4.5
4.0
3.5
3.0
2.5
2.0
1.5
1.0
0.5
0.0
f1 (ppm)

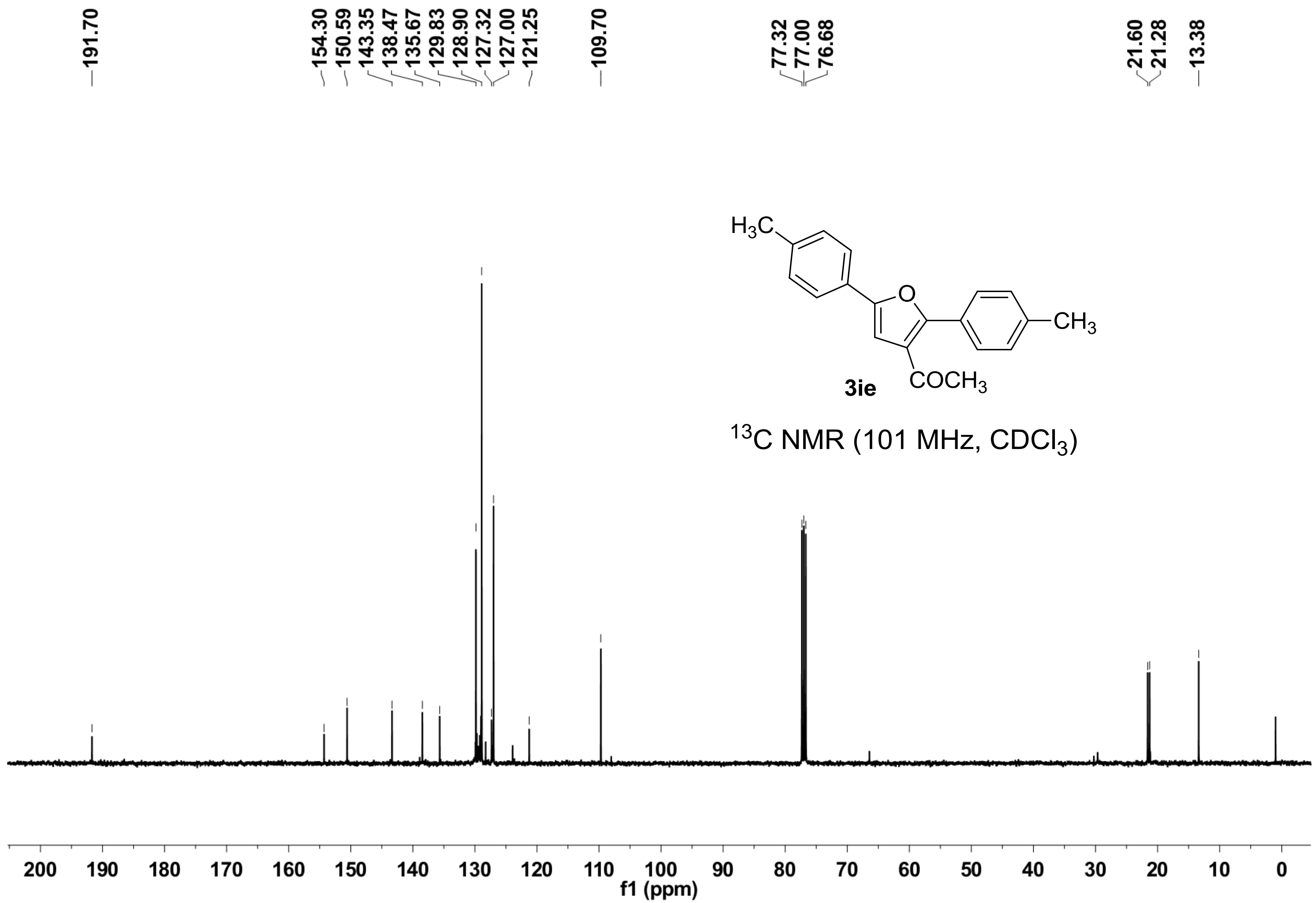
191.70
154.30
150.59
143.35
138.47
135.67
129.83
128.90
127.32
127.00
121.25
109.70
77.32
77.00
76.68
21.60
21.28
13.38
H_3C
O
CH_3
$COCH_3$
3ie
^{13}C NMR (101 MHz, $CDCl_3$)
200 190 180 170 160 150 140 130 120 110 100 90 80 70 60 50 40 30 20 10 0
f1 (ppm)
